# Rapid and Noninvasive Early Detection of Lung Cancer by Integration of Machine Learning and Salivary Metabolic Fingerprints Using MS LOC Platform: A Large‐Scale Multicenter Study

**DOI:** 10.1002/advs.202416719

**Published:** 2025-05-14

**Authors:** Shuang Lin, Runlan Yan, Junqi Zhu, Bei Li, Yinyan Zhong, Shuang Han, Huiting Wang, Jianmin Wu, Zhao Chen, Yuyue Jiang, Aiwu Pan, Xuqing Huang, Xiaoming Chen, Pingya Zhu, Sheng Cao, Wenhua Liang, Peng Ye, Yue Gao

**Affiliations:** ^1^ Department of Thoracic Surgery The First Affiliated Hospital School of Medicine Zhejiang University Hangzhou 310006 China; ^2^ Department of Geriatrics Zhejiang Key Laboratory of Traditional Chinese Medicine for the Prevention and Treatment of Senile Chronic Diseases, Affiliated Hangzhou First People’s Hospital School of Medicine Westlake University Hangzhou 310006 China; ^3^ Respiratory and Critical Care Medicine Department Tongde Hospital of Zhejiang Province Hangzhou 310012 China; ^4^ Pengbu Subdistrict Community Healthcare Center Shangcheng District Hangzhou 310000 China; ^5^ Department of Thoracic Oncology and Surgery China State Key Laboratory of Respiratory Disease & National Clinical Research Center for Respiratory Disease The First Affiliated Hospital of Guangzhou Medical University Guangzhou 510000 China; ^6^ Lab of Nanomedicine and Omic‐based Diagnosis Department of Chemistry Zhejiang University Hangzhou 310058 China; ^7^ Department of Thoracic Surgery Sir Run Run Shaw Hospital School of Medicine Zhejiang University Hangzhou 310016 China; ^8^ Department of Internal Medicine the Second Affiliated Hospital Zhejiang University School of Medicine Hangzhou 310058 China; ^9^ Respiratory and Critical Care Medicine Department Affiliated Hospital of Hangzhou Normal University Hangzhou 310000 China; ^10^ Well‐healthcare Technologies, Co., Ltd. Hangzhou 310012 China; ^11^ Department of General Surgery Sir Run Run Shaw Hospital School of Medicine Zhejiang University Hangzhou 310016 China

**Keywords:** early detection, lung cancer, machine learning, MS LOC, salivary metabolic fingerprints

## Abstract

Most lung cancer (LC) patients are diagnosed at advanced stages due to the lack of effective screening tools. This multicenter study analyzes 1043 saliva samples (334 LC cases and 709 non‐LC cases) using a novel high‐throughput platform for metabolic fingerprint acquisition. Machine learning identifies 35 metabolic features distinguishing LC from non‐LC subjects, enabling the development of a classification model named SalivaMLD. In the validation set and test set, SalivaMLD demonstrates strong diagnostic performance, achieving an area under the curve of 0.849‐0.850, a sensitivity of 81.69–83.33%, and a specificity of 74.23–74.39%, outperforming conventional tumor biomarkers. Notably, SalivaMLD exhibits superior accuracy in distinguishing early stage LC patients. Hence, this rapid and noninvasive screening method may be widely applied in clinical practice for LC detection.

## Graphical abstract

Salivary metabolic signatures associated with lung cancer are systematically characterized through a self‐developed high‐throughput mass spectrometry Lab‐on‐a‐Chip platform. Utilizing these metabolic fingerprints, SalivaMLD, an integrated machine‐learning‐driven classification model is developed and demonstrates superior performance in detecting lung cancer compared to conventional tumor biomarkers. This novel approach holds significant potential for clinical translation as a rapid and noninvasive diagnostic tool.

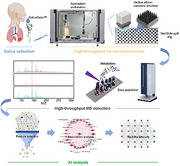

## 1 Introduction

Lung cancer (LC) is the leading cause of cancer‐related mortality globally, accounting for more than 1.82 million new deaths in 2022.^[^ ^1^ ^]^ Most LC patients are diagnosed in advanced stages, primarily because early stage LC often presents with no key symptoms and effective screening methods are currently limited. Studies have reported that the 5 years survival rates for LC patients in stage IA and stage IV are 85% and 5%, respectively,^[^ ^2^ ^]^ demonstrating the importance of early LC screening and detection. Low‐dose computed tomography (LDCT), the most recommended LC screening method, has been shown to reduce LC mortality by 20% in clinical trials.^[^ ^3^, ^4^, ^5^, ^6^ ^]^ However, LDCT is associated with a high false‐positive rate of LC screening (96.4%), leading to unnecessary invasive diagnostic procedures, serious psychological pressure, and financial burden to patients.^[^ ^7^, ^8^ ^]^ At present, most suspected LC cases screened by LDCT require further diagnosis via invasive methods, such as bronchoscopy, transbronchial aspiration, and surgery. Other commonly used clinical methods for LC detection, including X‐ray examination,^[^ ^9^ ^]^ serum tumor biomarker testing,^[^ ^10^ ^]^ and sputum cytology examination,^[^ ^11^ ^]^ all exhibit low overall sensitivity, particularly in early stage LC. Therefore, an effective discrimination method for large‐scale early LC screening and detection is highly demanded.

“Omics” have been recently used for screening studies of LC. Compared with conventional serum tumor biomarkers, such as carcinoembryonic antigen (CEA), carbohydrate antigen 125 (CA125), neuron‐specific enolase (NSE), and squamous cell carcinoma associated antigen (SCC), omics data combined with statistical algorithms can balance sensitivity and specificity, and improve the overall detection accuracy.^[^ ^12^, ^13^ ^]^ Metabolomics, as one of the newly developed omics, holds particular promise for precise cancer detection due to its ability to amplify biological signals and to reflect “what is happening” in organism.^[^ ^14^ ^]^ Studies have shown that metabolic dysregulation, such as glycolysis,^[^ ^15^ ^]^ single‐carbon metabolism,^[^ ^16^ ^]^ and de novo synthesis of lipids, occur in LC cells.^[^ ^17^ ^]^ LC and healthy control (HC) can be distinguished with high sensitivity and specificity through the detection and analysis of metabolic molecules in plasma or serum.^[^ ^18^, ^19^, ^20^, ^21^ ^]^ However, serum collection is not completely noninvasive, which can cause pain at the injection site, especially in pain‐sensitive people, and raises the risk of infection.

As a natural filtrate of blood, saliva is an ideal biofluid for on‐demand collection due to its abundant daily secretion (500–1500 mL in humans), noninvasive, being safe, and cost‐effective.^[^ ^22^, ^23^ ^]^ It contains various metabolic features, including amino acids, organic acids, lipids, and hormones,^[^ ^24^, ^25^, ^26^ ^]^ the abundance of which can reflect the dynamic equilibrium among saliva, blood, and tissue microenvironment.^[^ ^27^ ^]^ As a result, salivary metabolic fingerprint provides a comprehensive perspective of salivary metabolite composition, capturing the oncometabolites originating from metabolic rewiring, and highlighting those altered pathways during metabolic reprogramming.^[^ ^24^, ^28^, ^29^ ^]^ Salivary metabolic signatures have been used for detection or screening of oral squamous cell carcinoma,^[^ ^30^ ^]^ gastric cancer,^[^ ^31^ ^]^ and head and neck cancer.^[^ ^32^ ^]^ More importantly, studies have revealed metabolic disturbances in the saliva of LC,^[^ ^33^, ^34^ ^]^ potentially attributable to smoking,^[^ ^35^ ^]^ disturbance of oral intestinal flora, and other factors.^[^ ^36^ ^]^ Previous research reported salivary metabolite panels capable of distinguishing LC patients from control participants with cross‐validated area under the curve (AUC) of 0.729–0.744,^[^ ^34^, ^37^ ^]^ though lacking verification in an independent test set. In our previous study, a panel of salivary metabolites was identified to distinguish LC and HC with sensitivity of 97.2% and specificity of 92% in a verification cohort.^[^ ^38^ ^]^ However, these findings remain preliminary and limited to laboratory settings without fully optimized protocols for industrial‐scale application. Moreover, current models were primarily developed in single‐center studies with limited sample sizes, compromising their generalizability due to metabolic heterogeneity influenced by different regions, dietary habits, and other environmental or lifestyle factors.

Nevertheless, the development of salivary metabolic fingerprints‐based LC screening methods faces several technical difficulties. First, the absence of standardized saliva collection procedures presents a significant limitation, despite various saliva collection devices offering nonstimulated and stimulated collection methods as discussed in the literature.^[^ ^39^ ^]^ More critically, there is a lack of high‐throughput detection platform to capture salivary metabolic fingerprints for large‐scale cohort. Among various mass spectrometry (MS) technologies,^[^ ^40^, ^41^ ^]^ matrix‐assisted laser desorption/ionization time‐of‐flight mass spectrometry (MALDI‐TOF‐MS) demonstrates high precision, high sensitivity, and high throughput, enabling the rapid analysis of complex biological samples within a few seconds per sample.^[^ ^42^ ^]^ However, the use of organic matrices in MALDI‐MS can produce substantial background interference in the metabolic molecular weight region (<600 Da), making it difficult to acquire stable salivary metabolic fingerprints. Besides, omics data obtained from the MALDI‐MS may contain “noisy” peaks, which reduce the robustness and diagnostic performance of the model. Machine learning (ML), a widely adopted artificial intelligence (AI) approach, provides powerful tools for big data processing, including feature selection, model building, and rapid result interpretation.^[^ ^43^ ^]^ ML is particularly well‐suitable for the construction of disease discrimination models based on omics data, reducing potential misdiagnosis of diseases related to human expertise.^[^ ^44^ ^]^ Further screening in ML methodology and ensemble can enhance model performance and robustness.^[^ ^44^ ^]^ Nonetheless, AI‐based metabolic signature identification and model construction for LC screening based on MALDI‐MS data remain insufficiently explored and warrant systematic investigation.

In this study, a platform for saliva sample collection and salivary metabolic fingerprint acquirement using SalivaGetin and MS Lab‐on‐a‐Chip (MS LOC) was established. A multicenter retrospective cohort involving 1043 samples from six hospitals was enrolled and their salivary metabolic fingerprints were obtained using the self‐developed platform. Subsequently, we employed an AI‐assisted feature selection methodology to identify salivary metabolic features associated with LC. A machine learning model for LC diagnosis called SalivaMLD was constructed using an ensemble voting strategy. This model exhibited desirable performance metrics, indicating its potential suitability for broad clinical applications in LC screening and diagnosis.

## 2 Results

### 2.1 Platform Establishment for Metabolic Fingerprint Capture and Saliva Sample Collection

**Figure 1:**
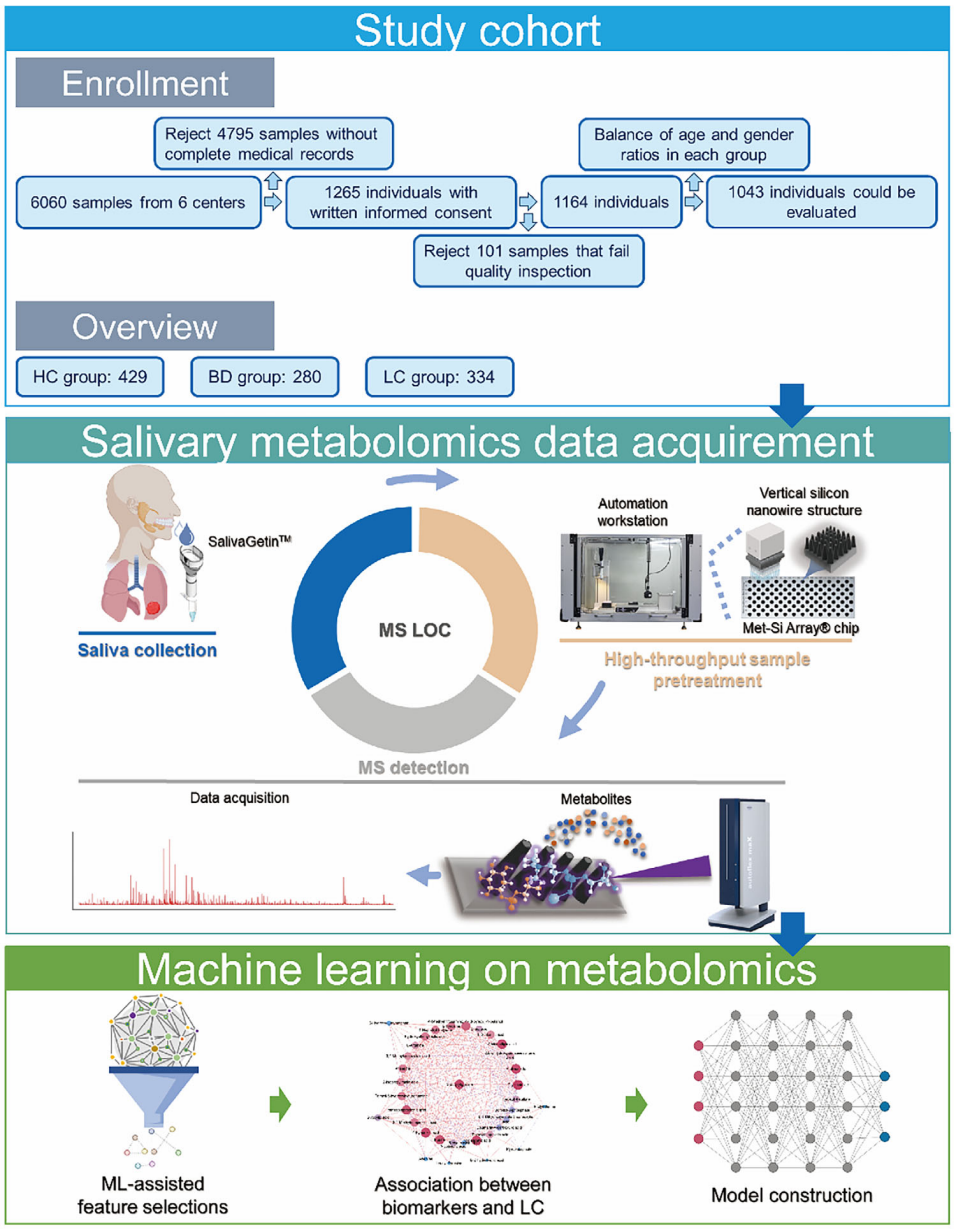
The process of acquisition and analysis of saliva metabolic fingerprints.

In this investigation, MS LOC consisted of nanomaterials array chip called Met–Si Array, automatic pretreatment protocol, and MALDI‐TOF‐MS instrument were established for the acquisition of salivary metabolic fingerprints (**Figure** **1**). Met–Si Array which possesses vertical silicon nanowires (SiNWs)^[^ ^45^, ^46^, ^47^ ^]^ displayed high sensitivity in the detection of small molecules with little background interference on the MALDI‐TOF‐MS. As shown in Figure S1 (Supporting Information), conventional organic matrices, such as α‐cyano‐4‐hydroxycinnamic acid and 2,5‐dihydroxybenzoic acid led to significant background interference in the low‐molecular‐weight region and produced uneven crystallization with minimal target molecule signals (Figure S1C,D, Supporting Information). By contrast, the proposed Met–Si Array chip, showed low background interferences and could significantly enhance the intensity and signal‐to‐noise (S/N) ratio of metabolite molecules, and promote repeatability (Figure S1B,E,F, Supporting Information). Additionally, a high‐throughput automatic protocol was developed for sample preparation (Figure 1). By programming, this protocol enabled high‐throughput preprocessing of 96 samples within 1 h. Also, the 4.5 mm spacing design of the arrays on Met–Si Array chip can perfectly match the automated spotting process. Less than 4 min was needed for spotting a batch of samples onto the wells of Met–Si Array. After sampling, the metabolic fingerprint of each sample could be automatically obtained via MALDI‐TOF‐MS within 2 s and the scanning of 108 wells on a Met–Si Array chip could be completed within 5 min (Figure 1). This entire protocol was evaluated for its stability. As shown in Figure S2 (Supporting Information), the intrabatch coefficients of variation (CVs) for three batches were 14.27%, 12.37%, and 11.55%, respectively, while the interbatch CV was 14.66%. Therefore, the entire process showed high‐throughput and high‐stable performance, reducing the error caused by manual operation.

Meanwhile, a novel saliva collection device named SalivaGetin was developed to facilitate stable and efficient saliva sampling. This device integrates a funnel, a dual‐layer filtration membrane, a barrel with plunger and a collection tube. The dual‐layer filtration membrane consists of a hydrophobic sieve plate and a low‐adsorption microporous membrane, enabling a stepwise removal of insoluble materials such as cellular debris, mucins, and oral bacteria. The pulling motion of flange allows filtered viscous samples to be drawn into the barrel more effectively (Figure S3A, Supporting Information). To evaluate the stability of SalivaGetin for salivary metabolomics, saliva samples from eight volunteers were collected using SalivaGetin, the Salivette‐assisted chewing method, and the conventional spitting method with centrifuge tubes (Figure S3B, Supporting Information). The saliva samples were either tested immediately or stored at 4 °C for 7 days before analysis. Mass spectrometric analysis of salivary metabolites tested immediately after sampling from these three methods, combined with the MS LOC platform, revealed a comparable mass spectrum (66–350 Da) (Figure S3C, Supporting Information). However, compared with other methods, saliva samples collected by SalivaGetin exhibited enhanced stability during storage with higher relevance (*R* > 0.8) (Figure S3D, Supporting Information). Overall, the proposed SalivaGetin combined with MS LOC platform enables rapid detection, high throughput, and high stability acquisition of salivary metabolic fingerprints, underscoring its potential as an optimal tool for salivary metabolomics analysis.

### 2.2 Study Cohort and Subject Characteristics

**Figure 2:**
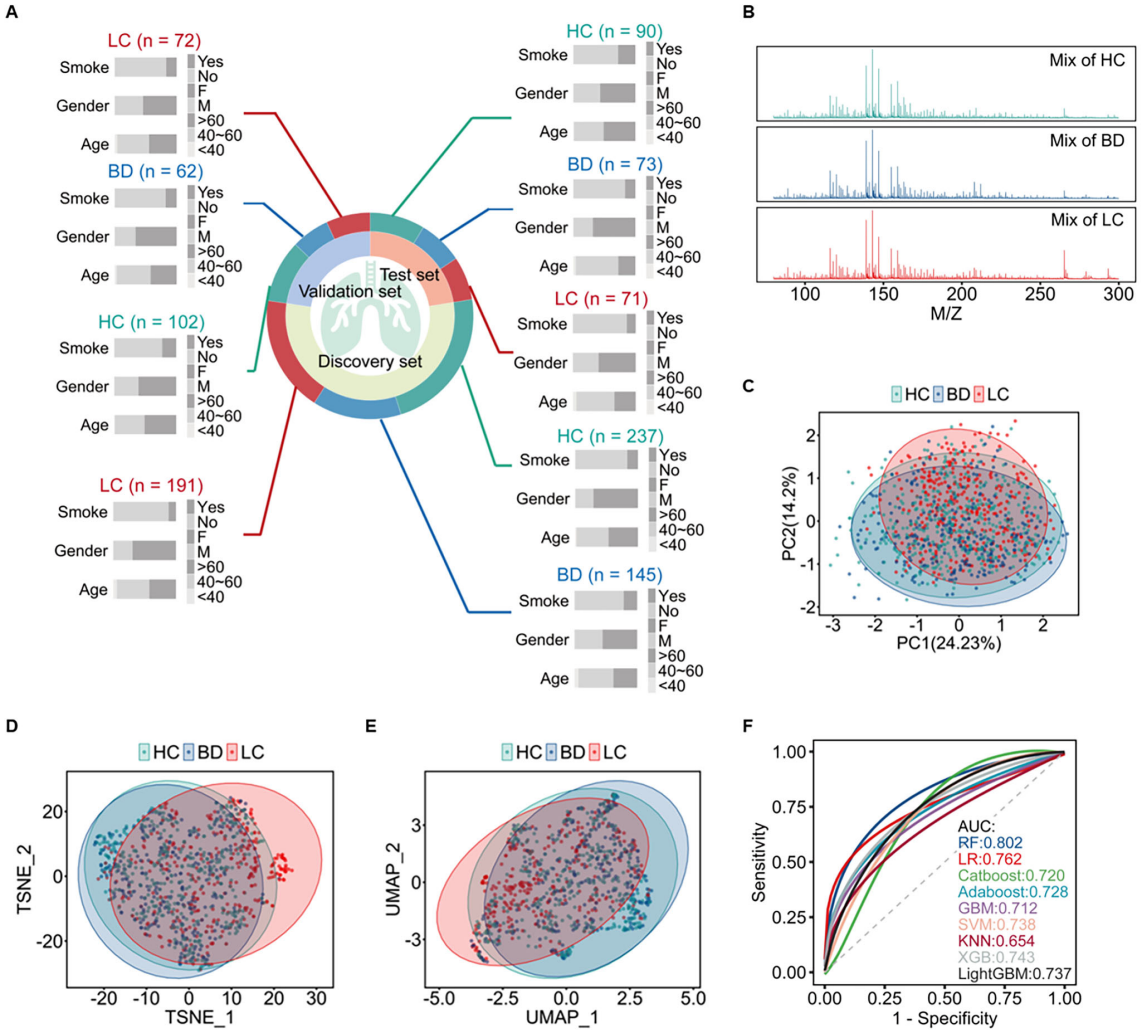
The overall differences in saliva metabolic fingerprints. A) Overview of clinical information distribution in the cohort. B) Salivary metabolic fingerprints of mixed samples in the HC, BD, and LC groups. C–E) 2D score plots of PCA, t‐SNE, and UMAP analyses among the HC, BD, and LC groups. All samples (*n* = 1043) in the cohort were used for PCA, t‐SNE, and UMAP analyses. F) Comparison of ROC curves for full‐feature models using nine algorithms on the validation set (*n* = 236). The 95% confidence interval (CI) for RF, LR, Catboost, Adaboost, GBM, SVM, *k*‐NN, XGB, and LightGBM were [0.740–0.865], [0.685–0.839], [0.640–0.799], [0.655–0.801], [0.634–0.789], [0.670–0.807], [0.581–0.728], [0.673–0.813], and [0.667–0.807], respectively.

In this study, 6060 volunteers were enrolled from six hospitals in China and their saliva samples were collected using the SalivaGetin sampling device. Among them, only 1265 individuals with written informed consent and complete medical records were kept. Following quality inspection and matching for gender and age between the LC group and the non‐LC group, a total of 1043 subjects were included in this analysis, comprising 429 HC cases, 280 benign diseases in lung (BD) cases, and 334 LC cases. Among the LC cases, 89.81% were diagnosed at early stages (Stage 0/I/II),^[^ ^48^ ^]^ with a predominant histologic subtype distribution (**Table** **1**). Lung adenocarcinoma (LUAD) accounted for the highest proportion (92.81%), further subclassified into adenocarcinoma in situ (AIS) (5.81%), minimally invasive adenocarcinoma (MIA) (32.58%), and invasive adenocarcinoma (IAC) (51.61%). SCC represented the second most frequent subtype (4.79%), whereas rare entities included large cell carcinoma (LCC) (0.60%) and small cell lung cancer (SCLC) (0.30%). Notably, three cases (0.90%) exhibited mixed histologic features consistent with adenosquamous carcinoma (ASC), a tumor type combining both LUAD and SCC components. Additionally, two cases (0.60%) remained unclassified due to ambiguous morphologic characteristics. The salivary metabolic fingerprints of the above 1043 subjects were acquired by MS LOC (Figure 1 and Table 1). Then, all samples were divided into discovery set, validation set, and test set. Discovery set was consisted of 237 HC cases, 145 BD cases, and 191 LC cases. Validation set contained 72 LC patients and 164 non‐LC controls including 102 HC cases and 62 BD cases, while the test set included 90 HC cases, 73 BD cases, and 71 LC patients (**Figure** **2**A). No significant age differences existed among the groups from the discovery set and validation set, except for the test set. However, gender distribution was significantly different among the groups in the discovery set. Additionally, the prevalence of family history of LC was not significantly different among all groups across the three subsets. Conversely, the ratio of smoking history and environmental exposure was imbalanced among these three groups from the discovery set and validation set, respectively (Table S1, Supporting Information). The influence of these clinical factors will be assessed in the subsequent cohort study.

**Table 1:** Demographic and clinical information for the study cohort.

Group	HC (*n* = 429)	BD (*n* = 280)	LC (*n* = 334)	Overall (*n* = 1043)
Age				
Mean (standard deviation)	58 (9)	57 (10)	58 (10)	58 (10)
Median [Min, Max]	60 [35, 79]	57 [36, 77]	58 [35, 80]	59 [35, 80]
Gender				
Female	281 (65.50%)	171 (61.07%)	213 (63.77%)	665 (63.76%)
Male	148 (34.50%)	109 (38.93%)	121 (36.23%)	378 (36.24%)
Smoking history				
No	341 (79.49%)	226 (80.71%)	290 (86.83%)	857 (82.17%)
Yes	88 (20.51%)	54 (19.29%)	44 (13.17%)	186 (17.83%)
Family history of lung cancer				
No	383 (89.28%)	250 (89.29%)	308 (92.22%)	941 (90.22%)
Yes	46 (10.72%)	30 (10.71%)	26 (7.78%)	102 (9.78%)
Environmental exposure				
No	366 (85.31%)	253 (90.36%)	307 (91.92%)	926 (88.78%)
Yes	63 (14.69%)	27 (9.64%)	27 (8.08%)	117 (11.22%)
Stage of lung cancer				
0	–	–	11 (3.29%)	–
I	–	–	280 (83.83%)	–
II	–	–	9 (2.69%)	–
III	–	–	12 (3.59%)	–
IV	–	–	3 (0.90%)	–
Unknown	–	–	19 (5.69%)	–
Subtypes				
LUAD	–	–	310 (92.81%)	–
SCC	–	–	16 (4.79%)	–
LCC	–	–	2 (0.60%)	–
ASC	–	–	3 (0.90%)	–
SCLC	–	–	1 (0.30%)	–
Unknown	–	–	2 (0.60%)	–
Classification of LUAD				
AIS	–	–	18 (5.81%)	–
MIA	–	–	101 (32.58%)	–
IAC	–	–	160 (51.61%)	–
Unknown	–	–	31 (10.00%)	–

### 2.3 ML Potential of Salivary Metabolic Fingerprints toward LC Identification

Over 60 000 data points were recorded in each saliva sample. The metabolic fingerprints of 138 quality control (QC) samples from 46 detection batches were also collected. MALDIquant package in R was employed for rapid preprocessing of the mass spectrometry data, resulting in the extraction of 646 distinct *m*/*z* signals. To assess data quality, the intensity distribution of all QC samples and individual ones were compared across 46 detection batches. Data showed that the corresponding intensity of the original mass spectrum signal was highly similar (Figure S4A, Supporting Information). What is more, the averaged interbatch CV of 646 signals for QC samples reached 11.74%, and almost all intrabatch CV below 10.0% (Figure S4B, Supporting Information). These findings indicate that the salivary metabolic fingerprints of the samples in this investigation were significantly stable and reliable. Considering that the samples came from multiple centers (Figure S5A, Supporting Information), unsupervised clustering analysis methods were conducted to explore the effects of center on salivary metabolic fingerprints. The analysis showed that sample clustering was unaffected by collection center, indicating minimal center‐specific variability (Figure S5B, Supporting Information). The mass spectrometry spectra from the mixed samples within each group are shown in Figure 2B. Unsupervised clustering analyses, including principal component analysis (PCA), t‐distributed stochastic neighbor embedding (t‐SNE), and uniform manifold approximation and projection (UMAP), consistently demonstrated that there was a certain degree of dispersion between the LC group and non‐LC groups (Figure 2C–E), suggesting that it was necessary to employ ML algorithms to enhance data interpretation and diagnostic performance. It was worth noting that there was minimal dispersion between the HC and BD groups.

Afterward, power analysis was performed on the salivary metabolic fingerprints to explore the minimum sample size required for ML. It was observed that a predicted power above 0.8 was achieved with a sample size of 105 per group, suggesting that the LC and non‐LC sample sizes in this cohort exceeded the necessary minimum (Figure S6, Supporting Information). To evaluate the efficacy of ML algorithms in distinguishing of LC from non‐LC based on salivary metabolic profiles, nine algorithms including logistic regression (LR), Catboost, gradient boosting machine (GBM), AdaBoost, lightGBM, *k*‐nearest neighbors (*k*‐NN), support vector machine (SVM), random forest (RF), and XGBoost, were applied to the discovery set utilizing all 646 *m*/*z* signals. Model construction and parameter tuning were constructed via tenfold cross‐validation. The performance of each model was then evaluated in the validation set. As shown in Figure 2F, models based on salivary metabolic fingerprints identified LC and non‐LC with AUC values of 0.654–0.802, thus highlighting the potential of ML in identifying LC from non‐LC samples. Given the variable performance across algorithms, further screening is necessary to optimize algorithm selection for feature selection and model construction.

### 2.4 Selection of LC‐Associated Metabolic Features Using an AI Strategy

**Figure 3:**
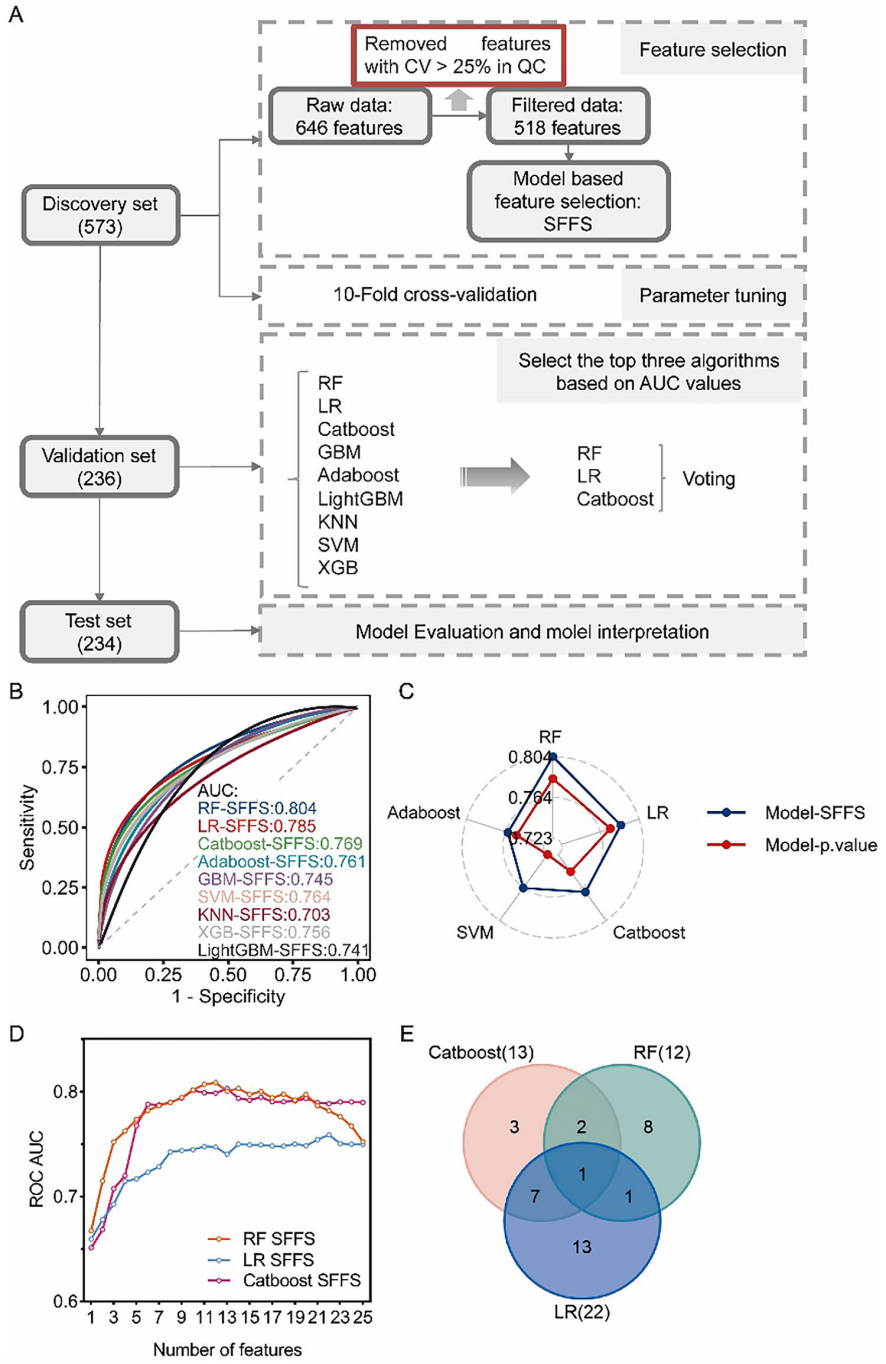
Performance comparison of feature selection algorithms. A) Flowchart of the machine learning modeling process. B) Comparison of ROC curves for nine SFFS‐based models on the validation set (*n* = 236). The 95% CI for or RF, LR, Catboost, Adaboost, GBM, SVM, *k*‐NN, XGB, and LightGBM was set at [0.739–0.868], [0.714–0.855], [0.700–0.838], [0.693–0.829], [0.678–0.812], [0.694–0.833], [0.629–0.776], [0.685–0.826], and [0.669–0.814], respectively. C) Radar chart comparing the AUC values of models on the validation set after feature selection using SFFS and *p*‐value filtering methods. D) Trend of the average AUC values in tenfold cross‐validation for the top three models during the SFFS feature selection process with different feature subsets. E) Venn diagram of features for the top three models based on AUC values.

LC‐associated metabolic features were identified by an AI‐based strategy (**Figure** **3**A). First, features with CV higher than 25% in the QC samples were excluded. Then, sequential floating forward selection (SFFS) method was applied to the discovery set to further refine the retained features. By iteratively adding or removing features based on the AUC value of models, the SFFS method effectively curated feature subsets (Figure S7, Supporting Information).^[^ ^49^ ^]^ For comparison, a traditional *p*‐value‐based feature selection method was used to extract features. Using different algorithms, nine SFFS‐based models were established with excellent performance, achieving AUC value of 0.703–0.804 when identifying LC and non‐LC in the validation set (Figure 3B). The top five algorithms from the SFFS models were also used to construct diagnostic models based on *p*‐value‐selected features. Across the top five algorithms, the AUC value of SFFS‐based models (0.761–0.804) consistently outperformed those of the *p*‐value‐based models (0.723–0.782) in the validation set (Figure 3C and Table S2 (Supporting Information)). Besides, the top five SFFS‐models performed better than that of all‐features‐based models (AUC = 0.720–0.802) (Figure 2F). It indicated that the subsets of features filtered by SFFS method outperform that of features screened by common *p*‐value‐based method or the full feature set when distinguishing between LC and non‐LC based on salivary metabolic fingerprints. Based on AUC rankings, the top three algorithms, including RF, LR, and CatBoost, were identified (Figure 3B). In these top three SFFS models, RF, LR, CatBoost, subsets of 12, 22, and 13 features were selected, respectively. The AUC values initially increased to an optimal point before exhibiting slight fluctuations or declines as additional features were incorporated in each algorithm (Figure 3D and Table S3 (Supporting Information)). Ultimately, a total of 35 features were obtained (Figure 3E).

**Figure 4:**
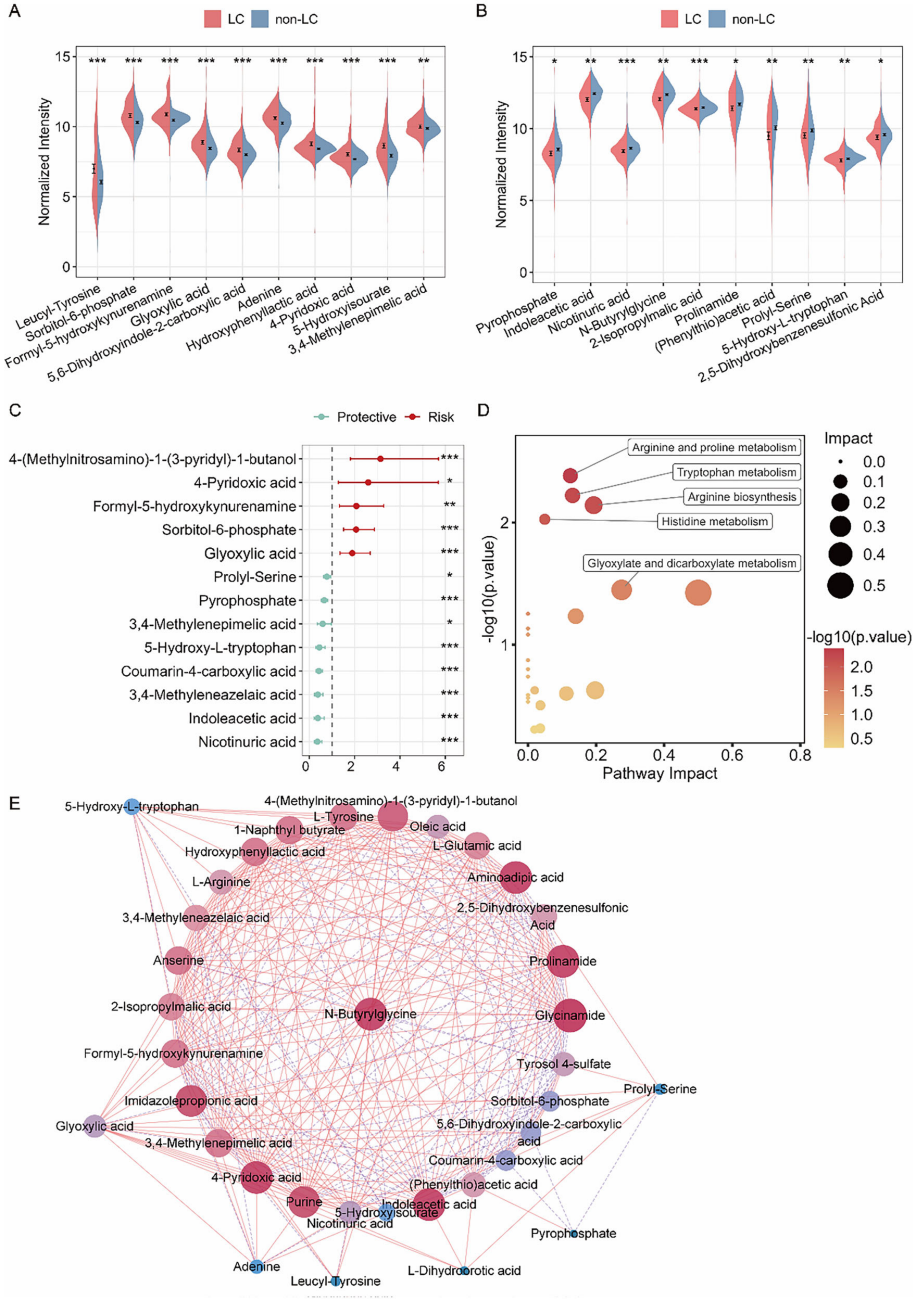
Functional characterization of the feature metabolites. A,B) Violin plot of upregulated features (A) and downregulated features (B) in LC versus non‐LC groups. Data were presented as mean ± standard error (SE). Difference of metabolites between LC and non‐LC groups were assessed with the Mann–Whitney *U* test, followed by FDR correction to obtain the *p*.value.adj (* <0.05, ** <0.01, *** <0.001). C) Forest plot of significant model features from OR analysis (95% CI) in the matched cohort for clinical characteristics including age, gender, smoking history, family history of lung cancer, and environmental exposure. The *p*.value was calculated using the Wald test (* <0.05, ** <0.01, *** <0.001). D) Bubble plot of pathway impact analysis for 35 features. Larger circles represent pathways containing more relevant metabolites, while deeper red coloring indicates greater statistical significance of the pathway's association. E) A correlation network of 35 model features, with correlations calculated using the Spearman method. The color and size of the nodes represent the number of edges, with red edges indicating positive correlation and purple edges indicating negative correlation. The above analysis was conducted using combined data from both the discovery and validation cohorts (*n* = 809).

The 35 features were annotated through MS/MS fragmentation, comparing the obtained *m/z* values of precursors and fragment ions with those of standard metabolites, and by searching Human Metabolome Database (HMDB) (Table S4, Supporting Information). Compared with the non‐LC group, 10 metabolites showed significantly upregulation, 10 were significantly downregulated (**Figure** **4**A,B), while another 15 showed no significant difference in LC patients. The distribution of these 35 features across the HC, BD, and LC groups was also illustrated using violin plots in Figure S8 (Supporting Information). Notably, the CV of all these 35 metabolites in the QC samples across 46 batches was below 25%, with the majority being under 15% (Figure S9, Supporting Information). Besides, most of these metabolites were not influenced by diet and circadian rhythm (Figure S10, Supporting Information), again confirming the stability of the entire protocol. Given the imbalanced distribution of age, gender, smoking history, and environmental exposure among HC, BD, and LC in certain datasets (Table S1, Supporting Information), odds ratio (OR) analysis was used to investigate the association of 35 metabolites with the occurrence of LC. It was observed that 13 metabolites were significantly correlated to the occurrence of LC after adjustment of clinical indicators (Figure 4C). It was also found that the correlation of content existed among metabolites (Figure 4D). The results of metabolic pathway analysis showed that several metabolic pathways, including arginine and proline metabolism, tryptophan metabolism, arginine biosynthesis, and histidine metabolism were disturbed in saliva of LC patients (Figure 4E). The disorder of metabolism was consistent with the tissue transcription sequencing results of LC from the TCGA database, due to the disturbance of transcriptional level of related metabolic enzymes, such as histidine ammonia‐lyase (HAL) (Figure S11, Supporting Information).

### 2.5 Construction and Evaluation of the Final Diagnostic Model for LC

**Figure 5:**
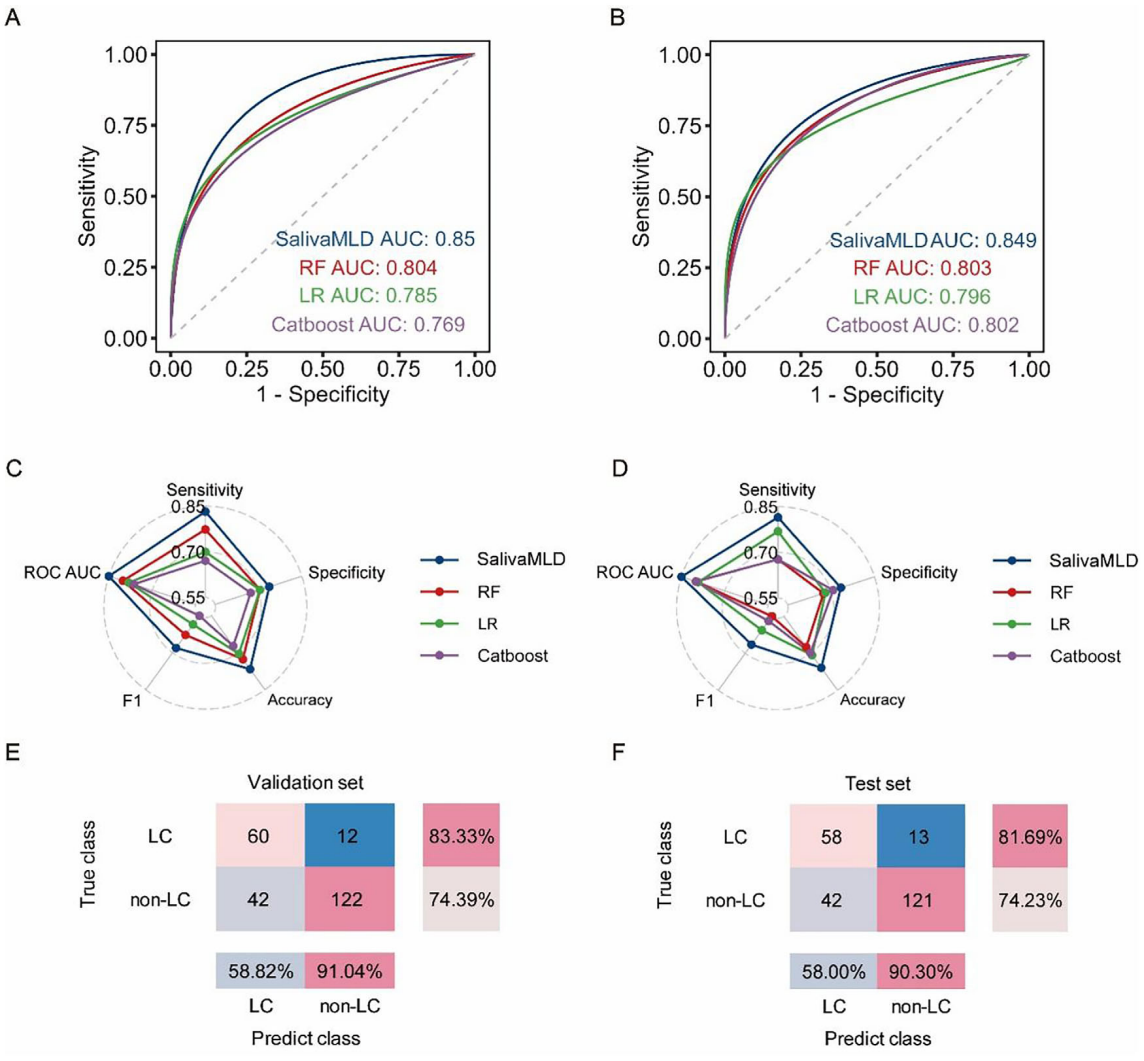
Construction and performance evaluation of the final machine learning ensemble model. A) Comparison of ROC AUC curves between the ensemble model and individual models on the validation set (*n* = 236). The 95% CI for SalivaMLD, RF, LR, and Catboost was set at [0.799–0.901], [0.739–0.868], [0.714–0.855], and [0.700–0.838], respectively. B) Comparison of ROC AUC curves between the ensemble model and individual models on the test set (*n* = 234). The 95% CI for SalivaMLD, RF, LR, and Catboost was set at [0.792–0.906], [0.741–0.866], [0.725–0.867], and [0.740–0.865], respectively. C,D) Radar charts comparing sensitivity, specificity, accuracy, ROC AUC, and F1 score between the final ensemble model and individual models on the validation (C) and test (D) sets. E,F) Confusion matrix of the final ensemble model on the validation (E) and test (F) sets.

To enhance mode performance, a final diagnostic model for LC, called SalivaMLD, was constructed using an ensemble strategy by voting on the top three SFFS models on the validation set (Figure 3A). Shapley Additive exPlanation (SHAP) analysis was adopted to explain the contribution of the 35 metabolites to the model predictions (Figure S12, Supporting Information). When identifying LC and non‐LC in the validation set, the AUC value of SalivaMLD reached 0.850, significantly higher than its basic models with AUC values of 0.769–0.804 in the validation set when differentiating LC and non‐LC (**Figure** **5**A). Moreover, sensitivity, specificity, accuracy, and F1 score of SalivaMLD were also higher than that of its basic models in the validation set (Figure 5C and Table S5 (Supporting Information)), demonstrating the advantages of the ensemble strategy by voting in LC identification. Specifically, the sensitivity, specificity, and accuracy of SalivaMLD reached 83.33%, 74.39%, 77.12%, respectively, in distinguishing LC and non‐LC in the validation set (Figure 5E and Table S5 (Supporting Information)). In the test set, SalivaMLD maintained high performance with an AUC of 0.849, a sensitivity of 81.69%, a specificity of 74.23%, and an accuracy of 76.50% (Figure 5B,F and Table S5 (Supporting Information)), further confirming its robustness in LC identification. SalivaMLD also outperformed the basic models in the test set (Figure 5D), indicating the benefit of combining algorithms for enhanced diagnostic accuracy. Moreover, SalivaMLD performed better than other algorithms – SFFS models in the test set, in terms of sensitivity, specificity, accuracy, and F1 score (Table S5, Supporting Information). Besides, in the validation set, the positive predictive value (PPV) and negative predictive value (NPV) for identifying LC and non‐LC were 58.82% and 91.04%, respectively. These results were consistent with those observed in the test set, where the PPV was 58.00% and NPV was 90.30% (Figure 5E,F). Given the class imbalance in the dataset with 334 LC cases and 709 non‐LC cases, class weighting was opted to address the dataset imbalance. Notably, similar performance was obtained, indicating that the performance was robust to class imbalance (Figure S13, Supporting Information). Moreover, the age distribution had no significant impact on the accuracy of SalivaMLD in the test set (Figure S14, Supporting Information). OR analysis also demonstrated that SalivaMLD was the independent diagnostic factor for LC, unaffected by multiple clinical factors, including age, gender, smoking history, family history of lung, and environmental exposure (**Table** **2**). These results highlight the reliability of SalivaMLD for LC screening.

**Table 2:** OR analysis of the SalivaMLD model's diagnostic performance before and after matching for clinical characteristics in the combined discovery and validation cohorts (*n* = 809). The *p*.value was calculated using the Wald test.

Index or model	Before matching			After matching		
	OR	95%CI	*p*.value	OR	95% CI	*p*.value
Age	1	[0.96–1.03]	0.88	0.72	[0.24–2.16]	0.56
Gender	0.70	[0.30–1.62]	0.40	0.84	[0.26–2.58]	0.76
Smoking history	0.44	[0.15–1.26]	0.13	0.69	[0.18–2.17]	0.54
Family history of lung cancer	0.71	[0.23–1.98]	0.52	1.11	[0.46–2.74]	0.82
Environmental exposure	0.29	[0.09–0.64]	8.7E−3	0.99	[0.96–1.03]	0.73
SalivaMLD	16.06	[7.91–35.04]	1.97E−13	15.59	[7.46–35.21]	2.87E−12

Further analysis of the SalivaMLD model's performance across different stages and subtypes of LC was conducted. The result demonstrated that SalivaMLD accurately identified 79.37–80.96% of early stage LC patients (Stage 0, Stage I, and Stage II) and 100.0% of late‐stage LC patients (Stage III and Stage IV) in both the validation and test sets (Table S6, Supporting Information). Specifically, the sensitivities of SalivaMLD in identifying LC patients in Stage 0, Stage IA, Stage IB, and Stage IIB were 75.00%, 82.35%, 83.33%, and 50.00% in the validation set, respectively. In the test set, SalivaMLD successfully distinguished 50.00% of Stage 0 patients, 81.48% of Stage IA, 66.67% of the Stage IB, and 100.00% of the Stage IIB cases. Interestingly, LUAD and SCC are the two dominant subtypes of LC in this study. SalivaMLD achieved accuracy rates of 83.58% and 82.54% accuracy for LUAD in the validation and test sets, respectively, and 75.00% and 80.00% for SCC in the validation and test sets (Table S7, Supporting Information). Additionally, for LUAD patients, the model demonstrated excellent performance in distinguishing AIS and MIA. These findings indicated that SalivaMLD exhibits strong diagnostic performance in identifying early LC.

### 2.6 Comparison of Performance between SalivaMLD and Traditional Tumor Biomarkers

**Figure 6:**
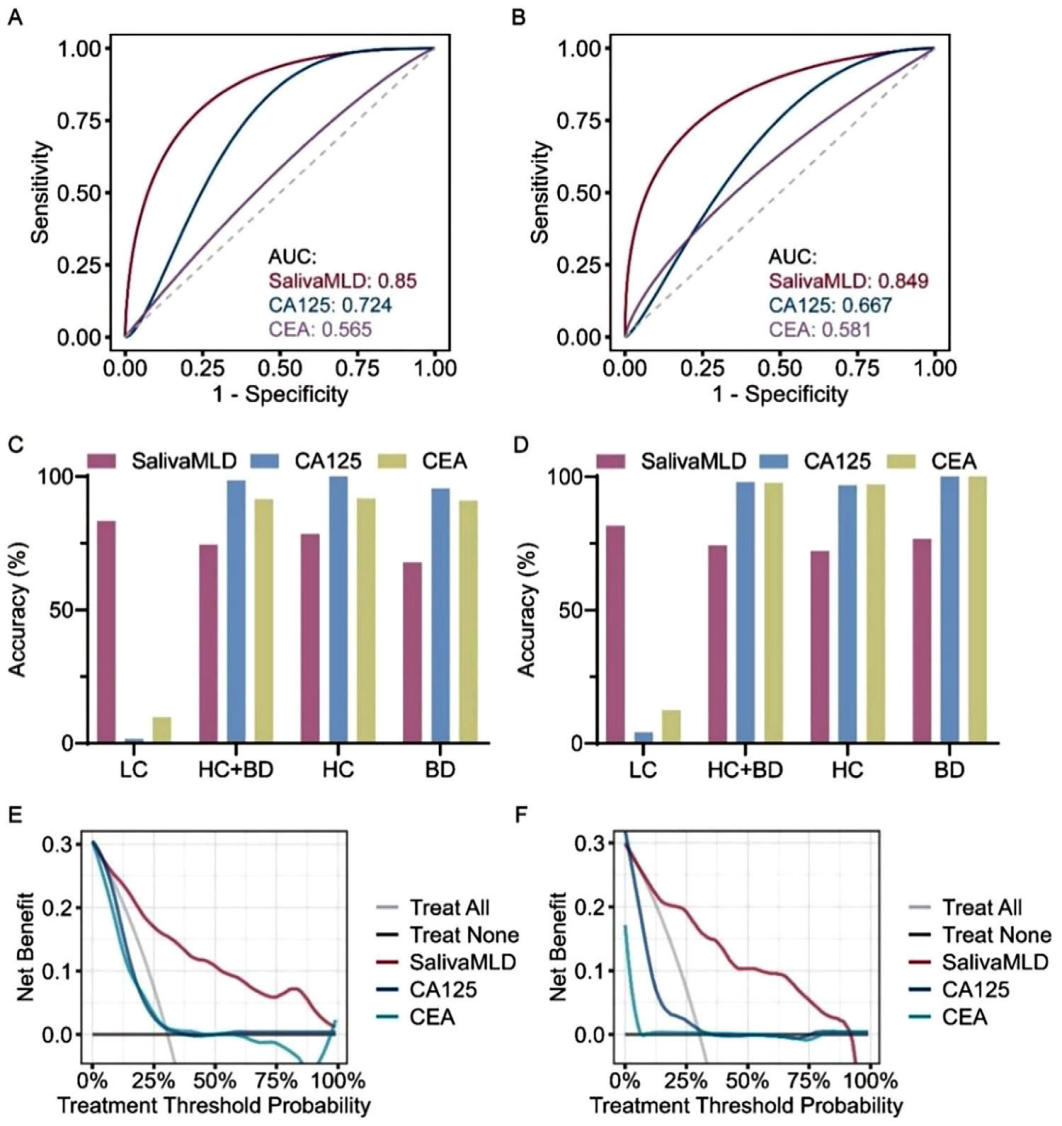
Comparison of model performance with tumor markers. A,B) Comparison of ROC AUC curves for SalivaMLD, CA125, and CEA on the validation (A) and test (B) sets. In the validation set (*n* = 236), the 95% CI for SalivaMLD, CA125, and CEA was set at [0.799–0.901], [0.637–0.811], and [0.476–0.653], respectively. In the test set (*n* = 234), the 95% CI for SalivaMLD, CA125, and CEA was [0.792–0.906], [0.572–0.763], and [0.488–0.674], respectively. C,D) Bar charts comparing the classification accuracy of SalivaMLD, CA125, and CEA for distinguishing LC, BD, and HC groups in the validation (C) and test (D) sets. E,F) Comparison of DCA curves for SalivaMLD, CA125, and CEA in the validation (E) and test (F) sets. DCA was performed using the dcurves package in R. Missing values of CEA and CA125 were filled with the median value.

Traditional blood‐based biomarkers for LC, such as CA125, NSE, CYFR A21‐1, and SCC, were detected in most of the LC groups and part of the non‐LC volunteers (Figure S15, Supporting Information). In identifying LC and non‐LC, SalivaMLD achieved higher AUC values of 0.850 and 0.849 in the validation and test sets, respectively, compared to CA125 (0.724, 0.667) and CEA (0.565, 0.581) (**Figure** **6**A,B). Though CA125 and CEA had a higher specificity of 98.57‐98.16%, 91.43–97.75%, respectively, higher than that of SalivaMLD (74.23–74.39%), SalivaMLD demonstrated superior sensitivity (81.69–83.33%) relative to CA125 (1.61–4.23%) and CEA (9.68–12.50%) (Figure 6C,D and Table S8 (Supporting Information)). When matched for similar specificity, sensitivity of SalivaMLD remained higher (81.69–83.33%) compared to CA125 (56.34–56.94%) and CEA (35.21–44.44%) in both datasets (Figure S16A, B and Table S9, Supporting Information). Meanwhile, CA125 and CEA had inferior specificity (22.09–25.00%) relative to SalivaMLD (74.23–74.39%) when sensitivities were aligned (Figure S16C,D and Table S9, Supporting Information). Moreover, NSE, CYFR A21‐1, and SCC arrived at 0.51–0.58, 0.63‐0.64, and 0.46–0.73 of AUC, which was all surpassed by SalivaMLD with AUC values of 0.849‐0.850 in both validation and test sets (Figure S17, Supporting Information). In addition, the clinical significance of SalivaMLD, CA125, and CEA was evaluated and compared using decision curve analysis (DCA). The results indicated that the area of positive net benefit for SalivaMLD was significantly greater than that of CA125 and CEA in both the validation and test sets (Figure 6E,F). These findings suggest that SalivaMLD may offer greater clinical advantages over conventional serum biomarkers in the diagnosis of LC patients.

Furthermore, the performance of combining SalivaMLD with CA125 or CEA was assessed to determine potential improvements in diagnostic accuracy. The addition of CA125 slightly increased the sensitivity of SalivaMLD accompanied with a decrease in specificity in the validation set (Figure S18A, Supporting Information). In the test set, the sensitivity of the combined SalivaMLD and CA125 model was comparable to that of SalivaMLD alone (Figure S18B, Supporting Information). The combination of SalivaMLD and CA125 could increase the AUC of the models from 0.850 to 0.895 in the validation set, while no change of AUC value was found in the test set (Figure S18E,F, Supporting Information). Similarly, combination of SalivaMLD and CEA produced comparable effects on the performance. Specifically, the participation of CEA did not simultaneously increase the sensitivity and specificity of SalivaMLD in identifying LC and non‐LC in the validation and test sets (Figure S18C,D, Supporting Information). Consequently, the addition of CEA slightly improved the AUC value from 0.849 to 0.887 in the test set, while has no impact on the validation set (Figure S18E,F). Overall, the data indicated that combining with traditional tumor biomarkers, such as CA125 and CEA, did not significantly enhance the diagnostic performance of SalivaMLD, including its sensitivity and specificity in distinguishing LC from non‐LC groups.

## 3 Discussion

Salivary metabolomics has potential in the diagnosis and monitoring of diseases, especially malignant tumors, including oral squamous cell carcinoma,^[^ ^30^ ^]^ gastric cancer,^[^ ^31^ ^]^ and head and neck cancer.^[^ ^32^ ^]^ However, different saliva collection methods and saliva metabolic fingerprint detection platforms have been adopted in different articles, and there is no unified standardized device. Salivette device is a widely used saliva collection device through mastication.^[^ ^31^ ^]^ However, chewing stimulation accelerates the secretion rate of saliva, which affects the abundance of metabolites, such as choline and taurine, in saliva.^[^ ^31^ ^]^ In this study, we developed a novel saliva collection device named SalivaGetin, which can collect unstimulated whole saliva. This device is consisted of a dual‐layer filtration membrane and a barrel with plunger, which facilitate saliva sample collection and preservation. Besides, SalivaGetin‐assisted sampling not only mitigates issues related to bacterial contamination and mucin aggregation that may impact sample stability in the spitting method, but also prevents artifacts associated with Salivette‐based methods, such as unintended saliva stimulation or material leaching from the swab. Totally, SalivaGetin‐assisted method standardizes the saliva collection process and can reduce variability. Besides, MS LOC platform consisted of Met–Si Array, an automatic pretreatment protocol and MALDI‐TOF‐MS was also established in this study. It offers advantages such as high sensitivity, simplified sample pretreatment, high throughput, and robust reliability. Based on the integration of SalivaGetin and MS LOC system, stable metabolic fingerprints of saliva samples were obtained and 646 m/z signals were detected in saliva, exceeding the 626 salivary features previously reported by Song et al.^[^ ^30^ ^]^ Despite previous studies have reported that salivary metabolic extracts can be obtained through simple dilution or by using cutoff filters,^[^ ^50^, ^51^ ^]^ our approach ensures greater stability and process control, as well as minimizing interference from salts and proteins in saliva. Moreover, this optimized workflow could complete automatic pretreatment, spotting, and analysis for a single batch within 2 h. Consequently, this platform, which performs excellently in detection speed, throughput, sensitivity, and stability, has potential to be applied in clinical to diagnosis and monitoring for diseases.

Herein, unsupervised clustering analysis revealed the different metabolic fingerprints between LC and non‐LC group, while demonstrating remarkable metabolic similarity between HC volunteers and BD patients, consistent with previous reports.^[^ ^52^ ^]^ Metabolomics generates vast amounts of data, and ML has emerged as a powerful tool for extracting clinically relevant patterns from these complex datasets, enabling the development of high‐performance models for clinical screening and detection. For instance, ML of salivary metabolic fingerprints can accurately distinguish oral squamous cell carcinoma and premalignant lesions with 86.7% accuracy.^[^ ^30^ ^]^ The integration of ML and serum metabolic patterns performed perfectly in identifying gastric cancer with an AUC value of 0.876–0.979.^[^ ^53^ ^]^ In this study, nine ML algorithms using all salivary metabolic features yielded moderate LC identification performance with an AUC value of 0.654–0.802 in the validation set. However, all‐features‐based ML models face inherent limitations in identifying biologically meaningful features, due to the potential noise from redundant or low‐contributing features.^[^ ^54^ ^]^ To address this, various features selection methods, such as the filtering, embedded, and wrapper methods, have been developed.^[^ ^55^ ^]^ The *p* value filtering method, which selects features based solely on their independent relationship with the target variable, may be less effective for complex datasets. By contrast, AI‐based feature selection strategy can mitigate this limitation.^[^ ^56^ ^]^ In this investigation, SFFS, which focuses on interactions between features and overall model performance, was employed to reveal LC‐associated salivary metabolic features. The subsets identified by SFFS exhibited superior performance (AUC = 0.703–0.804) compared to *p*‐value‐based subsets (AUC = 0.670–0.782) and the full feature set (AUC = 0.654–0.802) in distinguishing LC from non‐LC in the validation set. The enhanced diagnostic performance of the SFFS‐selected feature subsets highlights two key advantages: it effectively eliminates irrelevant variables while preserving important biomarker relationships, and it prioritizes optimizing model performance rather than focusing solely on the significance of individual features.

The three optimal SFFS‐derived models collectively identified 35 LC‐associated metabolic features. Beyond differences in metabolite abundance, the correlation between metabolites plays an important role in disease progression.^[^ ^57^ ^]^ Notably, only 20 features exhibited significant differences between LC and non‐LC groups with adjusted *p* values (*p*.value.adj) < 0.05, while all 35 metabolites demonstrated substantial intercorrelation. These metabolites were correlated at various levels and involved in pathways such as arginine and proline metabolism, tryptophan metabolism, arginine biosynthesis, and histidine metabolism, likely due to metabolic gene expression imbalances, such as HAL. Previous studies have shown that arginine and proline metabolism contribute to redox balance in esophageal squamous cell carcinoma,^[^ ^58^ ^]^ with proline biosynthesis overactivation leading to cancer‐associated fibroblast activation and tumor growth.^[^ ^59^ ^]^ Additionally, depletion of environmental arginine can impair T cell receptor signaling and T cell proliferation, inhibiting antitumor immunity.^[^ ^60^ ^]^ Disturbance of tryptophan metabolism can alter the balance between regulatory T cells and T_eff_ cells.^[^ ^61^ ^]^ Gut tryptophan metabolism impairment can activate the aryl hydrocarbon receptor and promotes liver cancer initiation.^[^ ^62^ ^]^ Moreover, after adjusting for clinical variables (gender/age) which effects metabolic profiles,^[^ ^63^, ^64^ ^]^ 13 of 35 metabolites remained significantly correlated with LC occurrence. This dual validation, both statistical significance and biological coherence, demonstrates AI‐driven feature selection's capacity to uncover latent pathophysiological networks. Unlike conventional differential analysis, SFFS identifies features that not only reflect significant differences but also reveal the underlying correlation among metabolites. These correlations likely reflect metabolic disorders associated with the onset and progression of LC, potentially through their impact on the tumor microenvironment and antitumor immunity, as observed in the saliva of LC patients. These characteristics make SFFS particularly well‐suited for clinical biomarker discovery, where complex interactions between features may underlie disease processes.

Although ML performs excellently in building models based on omics data, each algorithm has distinct strengths and limitations due to theoretical differences. Ensemble of different algorithms can enhance model performance and robustness.^[^ ^44^ ^]^ In this study, a diagnostic model for LC, SalivaMLD, was constructed using an ensemble strategy by voting the top three different algorithm‐based SFFS models. In the validation set, SalivaMLD identified LC and non‐LC with AUC value of 0.850, sensitivity of 83.33%, specificity of 74.39%, and accuracy of 77.12%. SalivaMLD also performed excellently with an AUC value of 0.849, a sensitivity of 81.69%, a specificity of 74.23%, and an accuracy of 76.50% in the test set. Besides, the performance of SalivaMLD was better than that of its basic models and other algorithm‐based SFFS models in the validation and test sets, confirming the preponderance of the voting ensemble strategy in the improvement of diagnostic performance for LC detection. Furthermore, it was observed that SalivaMLD was the diagnostic factor for LC independently of multiple clinical factors. It indicated SalivaMLD is robust and perfect in identifying LC from non‐LC cases. Moreover, other researchers have also established models to identify LC patients from controls based on salivary metabolites with AUC values of 0.729–0.744 in the training dataset,^[^ ^34^, ^37^ ^]^ without verification in the test set. It indicates that SalivaMLD performs better and is more credible. Given the substantial metabolic heterogeneity in LC,^[^ ^65^, ^66^ ^]^ this large‐scale, multicenter study recruited 1043 volunteers across six centers, affirming the broad applicability and significant potential of SalivaMLD for clinical use.

SalivaMLD also shows promising potential for early stage LC detection, which is crucial for reducing mortality.^[^ ^67^ ^]^ In this study, 79.37–80.96% of early stage LC patients (Stages 0–II) were accurately identified by SalivaMLD in the validation and test sets. Detecting LC at Stage I is important because patients treated at this stage have over 88% 10 years survival rates.^[^ ^68^ ^]^ While SalivaMLD exhibited reduced sensitivity in Stage 0 (75.00% in the validation set and 50.00% in the test set) compared to Stage I (82.46% and 80.00%, respectively), this is likely because these very early “precancer” lesions^[^ ^69^ ^]^ produce fewer detectable metabolic changes in saliva. Additionally, the small number of Stage 0 cases in our study (4 cases in the validation set and 2 cases in the test set) might influence the evaluation for the diagnostic performance. Importantly, the noninvasive nature of saliva‐based SalivaMLD screening allows for safe annual monitoring. This means that even if Stage 0 lesion is missed initially, it can still be caught at Stage I in subsequent screenings. For advanced LC patients (Stages III‐IV), 100% were all identified by SalivaMLD, though these results need confirmation with larger patient groups. Besides, the SalivaMLD model worked best for LUAD, which is the most common subtype of LC cases worldwide or China.^[^ ^70^ ^]^ It correctly identified 82.54–83.58% of LUAD cases, including early subtypes like AIS and MIA (75.00–83.33%) and later IAC types (83.33–85.37%). For SCC, another LC subtype, the detection rate was 75.00–80.00%, but this result was less certain due to the small number of SCC cases studied. Collectively, these results showed that SalivaMLD has strong diagnostic performance, especially for early stage LUAD. However, future studies should include more patients across all cancer stages (especially Stage 0 and late‐stage) and subtypes (like SCC, LCC, and SCLC) to confirm these results and ensure the test works equally well for all LC types.

CA125 and CEA are common serum tumor biomarkers for LC, with an AUC value of 0.519–0.752.^[^ ^71^ ^]^ In this study, SalivaMLD demonstrated statistically superior performance with an AUC value of 0.849‐0.850 relative to that of CA125 (AUC = 0.667–0.724) and CEA (ACU = 0.565–0.581) in identifying LC and non‐LC in the validation and test sets. SalivaMLD also exhibited notably higher sensitivity of 81.69–83.33% than CA125 (1.61–4.23%) and CEA (9.68–12.50%), calculated based on clinical cutoff values in both sets. Besides, SalivaMLD also exhibited a higher sensitivity than CA125 and CEA at comparable specificity levels. Furthermore, SCC, CYFRA21‐1, and NSE showed lower AUC values of 0.46–0.73, 0.63‐0.64, 0.51–0.58 than SalivaMLD in differentiating LC from non‐LC. These results highlight SalivaMLD, which is based on salivary metabolic fingerprints, as a more effective tool than conventional tumor biomarkers in identifying LC and non‐LC cases. It is also suggested that SalivaMLD may be a valuable complementary method for LC screening in individuals, who are negative in serological biomarkers tests. Notably, integrating CA125 or CEA with SalivaMLD via weighted voting did not improve performance, including its specificity in identifying LC. While the weighted voting method holds potential for optimizing integrated models like SalivaMLD compared to its basic models, its application in the integration of conventional serum biomarkers and SalivaMLD was limited, probably due to the partial biomarker data availability. Therefore, future large‐scale validation efforts will prioritize recruitment of patients with fully characterized tumor marker status to more accurately evaluate the potential of this integrated diagnostic approach.

Serum metabolite panels have shown high diagnostic potential for differentiating LC from controls, achieving a *C*‐statistic of 0.848^[^ ^18^ ^]^ or sensitivities of 70–90% and specificities of 90–93%.^[^ ^41^ ^]^ Although blood collection is minimally invasive, there are still some factors that limit its wide application. Blood collection requires medical personnel, which can be inconvenient and may bring the risk of infectious disease transmission due to hospital gatherings. By contrast, saliva and urine sampling are noninvasive, can be self‐administered at home, and allow for more frequent sampling over a short period of time. Meanwhile, a multicenter study showed that metabolic biomarkers in urine samples can identify LC with an AUC value of 0.78.^[^ ^21^ ^]^ However, our model SalivaMLD, which was built on salivary metabolic fingerprints, with an AUC value of 0.849‐0.850, is superior to the above urinary metabolite panel in identifying LC. Besides, mature sample collection method and standardized metabolic patterns acquisition platform for both urine and salivary has not yet been established. In this study, standardized procedures (seen in the Experimental Section) based on SalivaGetin for saliva sample collection and MS platform called MS LOC, have been developed for the stable acquisition of salivary metabolic fingerprints. These advancements enable stable transport home‐collected saliva samples to laboratory for testing, presenting significant potential in regions with well‐established express transport systems, such as China. Of course, the strategy of this home‐sampling and laboratory‐testing approach for LC screening needs further investigation on a broader scale.

LDCT is currently the most recommended method for lung cancer screening. However, specific instruments and experienced operators are required to accomplish LDCT testing, which restricts accessibility, especially in economically underdeveloped areas. By contrast, SalivaMLD offers a high‐throughput, cost‐effective alternative for LC detection, eliminating the need for technical personnel and thus increasing accessibility for populations unable to undergo LDCT screening LDCT examination. Moreover, LC screening by LDCT is mainly performed in high‐risk populations. Some people may not be aware of the severe risk factors of LC, such as occupational exposure history, and chronic lung disease history, while other risk factors of LC have not been discovered. The proposed SalivaMLD can facilitate LC screening, identify high‐risk populations for LDCT examination, and improve the allocation of clinical resources. Furthermore, LDCT screening has a high false‐positive rate of LC (96.4%),^[^ ^7^, ^8^ ^]^ making the differentiation of benign and malignant pulmonary nodules a major clinical difficulty. In this cohort, the non‐LC group included both HC and BD cases, which contained the volunteers with benign nodules. It is suggested that the integration of salivary metabolic fingerprints obtained by MS LOC, and ML could potentially identify benign and malignant lung nodules, which needs to be explored. Besides, while SalivaMLD demonstrates moderate specificity (74.23–74.39%), its current performance profile suggests opportunities for optimization through multimodal integration. We hypothesize that the combination of LDCT and SalivaMLD may improve the specificity, making the examination more specificity, which needs to be confirmed in the future.

## 4 Conclusion

In summary, our multicenter study utilizing a novel integrated saliva sampling device coupled with the MS LOC technology successfully established salivary metabolic fingerprints for 1043 participants, including LC, HC, and BD cases. AI‐assisted analysis revealed significant dysregulation of multiple metabolic pathways in the saliva of LC patients. Through ML optimization, we developed the SalivaMLD model, which demonstrated superior diagnostic performance compared to conventional tumor markers including CA125 and CEA. The model achieved AUC values of 0.849‐0.850, sensitivity of 81.69–83.33%, and specificity of 74.23–74.39% in validation and test cohorts. Notably, SalivaMLD exhibited enhanced diagnostic capability for early stage LC detection, suggesting clinical potential for early intervention. However, this study has several limitations that warrant improvement. First, partial unavailability of tumor biomarker records in some volunteers constrained comprehensive comparative analysis, though our observed biomarker performance aligned with the reported values.^[^ ^71^ ^]^ Further research is also needed to evaluate the synergistic effects between SalivaMLD, serum biomarkers, and LDCT imaging on the diagnostic performance for LC detection with complete multiomics datasets. Second, while the current study successfully established SalivaMLD's diagnostic efficacy for early stage LUAD, future validation across all TNM stages and histological subtypes would strengthen the model's pan‐cancer applicability. Third, although we implemented standardized collection protocol demonstrating excellent metabolite stability, further investigation is needed to characterize potential confounding factors including long‐term storage effects and lifestyle influences. Despite these limitations, this study demonstrates the feasibility of salivary metabolic fingerprints coupled with AI strategy for noninvasive LC screening in the real world.

## 5 Experimental Section

### Study Design

A total of 1043 volunteers were enrolled from six hospitals in China (Affiliated Hangzhou First People's Hospital, School of Medicine, Westlake University; the First Affiliated Hospital, School of Medicine, Zhejiang University; the First Affiliated Hospital of Guangzhou Medical University; Affiliated Hospital of Hangzhou Normal University, Hangzhou; Sir Run Run Shaw Hospital, School of Medicine, Zhejiang University; the Second Affiliated Hospital Zhejiang University School of Medicine). Saliva samples were retrospectively analyzed in a cohort comprising 334 LC patients, 280 patients with BD, specifically, benign lung lesions, and 429 HC. These saliva samples were collected between June 2021 and July 2022. The CT scans of healthy volunteers confirmed the absence of lung nodules. BD patients were clinically diagnosed. Benign pulmonary nodules in BD patients were confirmed via histologic examination or defined by one year volumetric stability, two years diameter stability, or resolution by CT scanning.^[^ ^72^ ^]^ LC patients were diagnosed and staged via histopathological examination, according to the International Association for the Study of Lung Cancer 8th TNM Tumor (tumor, node, and metastasis) classification. The exclusion criteria were as follows: 1) participants with a history of pulmonary infection within one month prior to enrollment; 2) pregnant or lactating women. Notably, all 334 LC patients, with no history of cancer, underwent saliva sampling before surgical resection or other therapeutic interventions. This study was approved by the Ethics committee of Affiliated Hangzhou First People's Hospital, School of Medicine, Westlake University (Ethical numbers IRB#2021‐20210401‐01, IIT‐20220112‐0006‐01). The basic clinical information, including tumor biomarkers, nodule/tumor tissue diameter, and pathological type, was also collected. All participants provided written informed consent.

### Saliva Sampling and Storage

Saliva samples were collected using a custom‐designed saliva collection device, SalivaGetin (patent application ZL 2020 2 1562927.1). Prior to saliva sampling, volunteers underwent oral preparation according to established protocols.^[^ ^38^, ^73^ ^]^ Briefly, saliva samples were collected between 7.30 and 11.30 am from hospitalized volunteers or individuals undergoing medical examination. To ensure sample consistency, participants were required to brush their teeth in the morning for saliva collection and to abstain from eating, drinking, brushing their teeth, or exercising for at least 1 h (typically 1‐2 h) prior to saliva collection. Smoking, oral nebulization, and the use of lipstick were also prohibited on the day of sampling. The volunteers rinsed their mouths thoroughly with purified water thrice, swallowed any residual water, and waited for 5 min before saliva collection to eliminate potential dilution effects from residual water. Then, ≈0.5 mL of unstimulated saliva was collected via passive drool in the funnel position of SalivaGetin. The saliva sample was then passed through two layers of membranes within the collection device to remove oral bacteria, mucins, cell debris, and other insoluble materials,^[^ ^39^ ^]^ and subsequently stored in the sterile tube kept on ice. QC samples were prepared by pooling equal volumes from 50 individual samples collected using the SalivaGetin device. All collected samples were stored at −80 °C until MS analysis. Additionally, unstimulated saliva were collected from eight volunteers using 50 cc polypropylene tubes and with the Salivette device (Sarstedt, Nümbrecht, Germany)^[^ ^39^ ^]^ for methodological comparison. Saliva samples from these eight volunteers were either immediately processed for MS detection or stored at 4 °C for 7 days before MS analysis to evaluate the impact of three different collection devices on the stability of salivary metabolome. Furthermore, saliva samples were collected from volunteers under varying conditions (morning fasting, 1 h postbreakfast, 2 h postbreakfast) and on consecutive days to verify the influence of temporal and dietary factors on salivary metabolites (detailed method could be found in the Supporting Information).

### Sample Pretreatment of Saliva

The salivary metabolome was extracted using a protein precipitation method^[^ ^74^ ^]^ on a 12‐channel automated liquid handing platform, ASSIST PLUS (INTEGRA, Switzerland), equipped with sample zone temperature control and chamber‐wide humidity regulation. Each batch included a total of 32 samples, consisting of 29 individual clinical samples and 3 QC samples, with each sample pretreated and prepared in triplicate following a randomized layout. Briefly, saliva samples stored at −80 °C were thawed on ice and arranged in rows of 12 samples in the cold zone (4 °C) of the platform. A 96‐well polymerase chain reaction (PCR) plate, preloaded with 20 µL of a mixture of acetonitrile/methanol (1:1, v/v) per well, was prepared as the reaction plate. Subsequently, 10 µL of saliva from each sample was transferred to the reaction plate via the ASSIST PLUS robotic arm followed by vortexing of the plate at 1500 rpm for 5 min. Then, the PCR plate was centrifuged at 2200 *g* for 5 min to isolate supernatant. The supernatant (12 µL) was transferred to a new PCR plate, followed by the addition of 4 µL ultrapure water. After thorough mixing, the resulting solution from each sample was automatically deposited onto the Met–Si Array chip (Well‐healthcare Technologies Co., Ltd., Hangzhou, China), which contained arrays of vertical SiNW structure.^[^ ^45^, ^46^, ^47^ ^]^ Samples were subsequently dried for 30–40 min under the controlled humidity of 40–50% in the liquid handing instrument. To validate the performance between the Met–Si Array chip and the organic matrix, a mixture of 5 amino acids (5AAs) and a mixture of 5AAs with saliva QC metabolic extract was prepared and analyzed, as detailed in the Supporting Information.

### Acquirement of Saliva Metabolic Fingerprints and Feature Annotation by MS LOC

Following sampling, the Met–Si Array chip was inserted into the autoflex Max MALDI‐TOF/TOF mass spectrometer (Bruker Daltonics Inc.) for acquisition of salivary metabolic fingerprints. The instrument was equipped with a 355 nm Nd:YAG laser, with a pulse energy < 500 µJ, a pulse width of <3 ns, and a laser frequency of 1000 Hz. The laser parameter was set at 4_large, yielding a laser spot size of 80–100 µm. Data were acquired with 1250 accumulated laser shots per sample using a Radom walk signal acquisition method. Saliva metabolome analysis was conducted in the reflectron negative ion mode, within a *m/z* range of 66–350, following MS calibration. Metabolic profiling of cohort samples was carried out in an automatic batch mode. MS/MS fragmentation of salivary features was performed on the autoflex maX MALDI‐TOF/TOF‐MS instrument. Metabolite feature annotation was achieved by comparing the acquired *m/z* values of precursors and fragment ions with standard metabolites, and by searching HMDB (http://www.hmdb.ca/). The relative error of *m/z* values of the precursors was set to 35 ppm.

### Data Processing and Comparative Analysis of Saliva Metabolic Fingerprints

In this study, the saliva metabolic fingerprints were preprocessed using the MALDIquant package in R, including steps such as smoothing, baseline correction, intensity normalization, alignment, peak detection, and peak binning. A total of 646 metabolite peaks were detected in the spectrum, which was expressed in over 80% of the samples (S/N > 5). Stability of the entire workflow was assessed by calculating the CV of QC samples. PCA, t‐SNE, and UMAP were used to evaluate group distributions. These analyses were implemented in R, using the prcomp function for PCA, the Rtsne package for t‐SNE, and the umap package for UMAP. Differential metabolites were assessed with the Mann–Whitney *U* test, followed by FDR correction to obtain the *p*.value.adj. Correlation analysis was conducted using Spearman's correlation via the psych package. Pathway analysis was performed using the MetaboAnalyst R package, while OR analysis using LR algorithm was applied to evaluate the impact of features. Furthermore, the transcriptomic data from tumors and normal tissues were downloaded from the publicly available TCGA database (https://www.cancer.gov/tcga) to investigate the LC‐associated metabolic dysregulation. To estimate the proper sample size and predictive power, machine‐learning‐based power analysis^[^ ^56^ ^]^ was applied in the discovery set. Detailed protocol for transcriptomic data analysis and power analysis could be found in the Supporting Information.

### Model Construction and Performance Assessment

The entire cohort of 1043 samples was divided into a discovery set, a validation set, and a test set in a ratio of ≈7:3:3. First, features with CV higher than 25% in the QC samples were excluded. Then, the SFFS method,^[^ ^54^ ^]^ based on nine algorithms including RF, LR, CatBoost, GBM, AdaBoost, LightGBM, *k*‐NN, SVM, and XGBoost, was used for feature selection in the discovery set. Afterward, model parameter tuning was conducted, leading to the development of nine classification models. These models were evaluated in the validation set using five performance metrics: sensitivity, specificity, accuracy, F1 score, and AUC. The top three models were then selected, and an ensemble strategy based on a voting mechanism was employed to build a classification model named SalivaMLD. Following the construction of the ensemble model, its performance was both assessed in the validation and test sets, and the SHAP method was applied to interpret the model, providing SHAP value distributions for each feature across the samples. Both the SFFS and SHAP analyses were implemented in Python (3.11). Receiver operating characteristic (ROC) analysis was conducted using the pROC package in R. The strategy of weighted voting was also applied to combine the detection values of tumor markers CA125 and CEA with the predicted probability values from the SalivaMLD model for prediction and classification. To assess the model's net benefit at various threshold probabilities, DCA was performed using the dcurves package in R. Detailed protocols for the SFFS strategy and ensemble voting could be found in the Supporting Information.

### Statistical Analysis

In this study, all statistical analyses were conducted using R software (http://www.R‐project.org, version 4.3.2). Total ion current method was used to normalize the raw MS data to mitigate the intersample intensity variance. Normality assessment of the data was completed using the Shapiro–Wilk test to guide analytical strategy selection. Nonparametric comparisons of continuous variables between groups were conducted using the Mann–Whitney *U* test with a two‐tailed evaluation, followed by FDR correction. For categorical variables including demographic parameters (age, gender) and clinical covariates (smoking history, family history of lung cancer, and environmental exposure), Chi‐square tests were used to assess significant differences between groups. The *p*.value for the OR analysis was calculated using the Wald test. In all statistical tests, a *p*.value or adjusted *p*.value less than 0.05 was considered statistically significant.

## Conflict of Interest

The authors declare no conflict of interest.

## Author Contributions

S.L., R.Y., and J.Z. contributed equally to this work. J.W., W.L., P.Y., and Y.G. conceived and designed the research. S.L., R.Y., and J.Z. performed research, analyzed data, and wrote the paper. J.Z., B.L., Y.Z., S.H., H.W., Z.C., Y.J., A.P., and X.H., included specimen. X.C., P.Z., and S.C. contributed new analytic tools and analyzed data.

## Supporting information



Supporting Information

## Data Availability

The data that support the findings of this study are available from the corresponding author upon reasonable request.
