# Supplementary material for: Rapid and Noninvasive Early Detection of Lung Cancer by Integration of Machine Learning and Salivary Metabolic Fingerprints Using MS LOC Platform: A Large‐Scale Multicenter Study
Source: Adv Sci (Weinh). 2025 May 14;12(22):2416719. doi: 10.1002/advs.202416719 (PMC12165017; doi:10.1002/advs.202416719)
Supplement: Supplementary file 1 — Supporting Information [file ADVS-12-2416719-s001.pdf]

ADVANCED SCIENCE
Open Access

Supporting Information

for *Adv. Sci.*, DOI 10.1002/advs.202416719

Rapid and Noninvasive Early Detection of Lung Cancer by Integration of Machine Learning and Salivary Metabolic Fingerprints Using MS LOC Platform: A Large-Scale Multicenter Study

Shuang Lin, Runlan Yan, Junqi Zhu, Bei Li, Yinyan Zhong, Shuang Han, Huiting Wang, Jianmin Wu, Zhao Chen, Yuyue Jiang, Aiwu Pan, Xuqing Huang, Xiaoming Chen, Pingya Zhu, Sheng Cao, Wenhua Liang, Peng Ye* and Yue Gao**

Supplementary Information

Rapid and Non-invasive Early Detection of Lung Cancer by Integration of Machine Learning and Salivary Metabolic Fingerprints using MS LOC Platform: a Large-scale Multi-center Study

Shuang Lin[b,1], Runlan Yan[a,1], Junqi Zhu[d,1], Bei Li[a], Yinyan Zhong[e], Shuang Han[a], Huiting Wang[c], Jianmin Wu[f], Zhao Chen[g], Yuyue Jiang[d], Aiwu Pan[h], Xuqing Huang[k], Xiaoming Chen[i,j], Pingya Zhu[i], Sheng Cao[i], Wenhua Liang[c,*], Peng Ye [b,*], Yue Gao[a,*]

a. Department of Geriatrics, Zhejiang Key Laboratory of Traditional Chinese Medicine for the Prevention and Treatment of Senile Chronic Diseases, Affiliated Hangzhou First People's Hospital, School of Medicine, Westlake University, Hangzhou, 310006, China.

b. Department of Thoracic Surgery, the First Affiliated Hospital, School of Medicine, Zhejiang University, Hangzhou, 310006, China.

c. Department of Thoracic Oncology and Surgery, China State Key Laboratory of Respiratory Disease & National Clinical Research Center for Respiratory Disease, the First Affiliated Hospital of Guangzhou Medical University, Guangzhou, 510000, China.

d. Respiratory and Critical Care Medicine Department, Tongde Hospital of Zhejiang Province, Hangzhou, 310012, China

e. Pengbu Subdistrict Community Healthcare Center, Shangcheng District, Hangzhou, 310000, China.

f. Lab of Nanomedicine and Omic-based Diagnosis, Department of Chemistry, Zhejiang University, Hangzhou, 310058, China.

g. Department of Thoracic Surgery, Sir Run Run Shaw Hospital, School of Medicine, Zhejiang University, Hangzhou, 310016, China.

h. Department of Internal Medicine, the Second Affiliated Hospital Zhejiang University School of Medicine, Hangzhou, 310058, China.

i. Well-healthcare Technologies, Co., Ltd., Hangzhou, 310012, China.

j. Department of General Surgery, Sir Run Run Shaw Hospital, School of Medicine, Zhejiang University, Hangzhou, 310016, China.

k. Respiratory and Critical Care Medicine Department, Affiliated Hospital of Hangzhou Normal University, Hangzhou, 310000, China.

Supplementary Methods

1. Saliva sampling for verification of temporal and dietary effects on salivary metabolites

Saliva samples of volunteers were collected using SalivaGetin^{TM} under different temporal and dietary conditions to investigate their effects on salivary metabolites. Firstly, a single volunteer collected the saliva samples under three conditions on the same day: morning fasting, 1 h post-breakfast and 2 h post-breakfast. For each condition, three replicate saliva samples were collected, generating a total of 9 MS spectra. Secondly, three volunteers were enrolled to collect the saliva samples at 1-h post-breakfast over three consecutive days. In each day, three separate saliva samples were collected per volunteer using different SalivaGetin^{TM} devices. 9 MS spectra per volunteer was acquired. Lastly, three volunteers collected their saliva samples at 9:00 am on two consecutive days: Time 1 (morning fasting on Day 1) and Time 2 (1-hour post-breakfast on Day 2). In each day/time, two separate saliva samples were collected per volunteer using two SalivaGetin devices, with triplicate pretreatment processes performed per sample. A total of 12 MS spectra per volunteer were generated for further analysis.

2. Preparation of QC+5AA

Five amino acids, including succinic acid, pyroglutamic acid, L-hisidine, cysteic acid, N-acetylhistidine were mixed and dissolved in a metabolite extract solution prepared from quality control (QC) saliva samples, yielding a final concentration of 0.033 μg/μL for each amino acid. This mixture, designated as “QC+5AAs” and the saliva QC metabolite extract alone (designated “QC”) were used as model analytes to evaluate the laser desorption/ionization (LDI) efficiency and stability of Met-Si Array, α-cyano-4-hydroxycinnamic acid (CHCA) and 2,5-dihydroxybenzoic acid (DHB). The metabolite extract solution derived from QC saliva samples was used to simulate a complex biological background.

3. LDI efficiency and stability comparison

The CHCA and DHB matrices were respectively prepared by dissolving each matrix powder in an acetonitrile/water mixture (ACN/H_2O, 1:1, v/v) to form supersaturated solutions. Both solutions were vortexed for 1 min and ultrasonicated for 10 min prior to use. For each sample analysis, 1 μL of QC or QC+5AAs solution and 1 μL of matrix solution were sequentially spotted onto the MALDI steel target plate and allowed to dry for 15 min at RT. QC and QC+5AA samples were also deposited onto the Met-Si Array for analysis. Each sample preparation method was tested with a total of six spots on either the MALDI steel target or the Met-Si Array, and analyses were performed in reflectron negative ion mode. Notably, the relative laser energy applied to the CHCA and DHB matrices (78%) was higher than that applied to the Met-Si Array (71.2%), to optimize ionization performance.

4. Protocol for Sequential Floating Forward Selection (SFFS)

In this study, the Sequential Floating Forward Selection (SFFS) method was employed for feature selection within a 10-fold cross-validation framework to ensure robust model evaluation. The feature selection process began with an empty feature subset, which was iteratively expanded by adding the best-performing feature based on its AUC value in each cross-validation fold. The AUC value across all folds was averaged to evaluate the performance of each feature subset. The floating mechanism was applied by removing the least useful features after each addition, ensuring an optimal feature set. This process was fully integrated with 10-fold cross-validation, where feature selection and model training were isolated to prevent data leakage and overestimation of performance. As a result, the feature subset selection was guided by accurate and generalizable performance metrics, enhancing the model's reliability and preventing overfitting.

5. Strategy for ensemble voting

In this study, the ensemble model leveraged three base models—Random Forest (RF), Logistic Regression (LR), and CatBoost. These models were trained on the discovery set using cross-validation to obtain their respective trained models. Once trained, each base model made predictions on the validation set, outputting predicted probability values. To combine these predictions into a final predicted probability, a weighted average soft voting strategy was employed. The weights for each model in the ensemble were optimized based on the AUC value of the ensemble model on the validation set. The optimization process involved exploring different weight combinations to identify the optimal weight distribution that maximized the AUC. Given three weights w_1, w_2 and w_3, each constrained to lie within the range of 0 to 1 and satisfying the condition $w_1 + w_2 + w_3 = 1$, the weights were discretized within specified ranges. This discretization produced multiple candidate weight combinations for evaluation. Each weight combination was applied to the ensemble, and the corresponding AUC value was calculated. After exhaustively evaluating all possible combinations, the one with the highest AUC was selected as the optimal weight distribution. This strategy effectively optimized the weight allocation, thereby enhancing the predictive performance of the ensemble model. Through iterative optimization, the final weight scheme was determined to maximize the ensemble's AUC, leading to improved overall model performance.

6. Power analysis

Power analysis is a critical methodological approach to determine the minimum sample size necessary for a study to detect true effects with adequate probability, typically set at ≥ 80%, under predefined effect sizes and significance levels ($\alpha = 0.05$). In conducting power analysis, four essential parameters must be defined: *effect size*, *significance level (α)*, *power*, and *degrees of freedom*.

Effect size, quantified using Cohen's f^2, reflects the magnitude of the hypothesized relationship

between variables. To calculate the effect size for a logistic regression model trained on the discovery set, the model was first fitted using predictors and a binary target variable through the glm function in R. Cohen's f^2 was then computed by comparing the R^2 values of the full model (with predictors) and a reduced model (intercept-only). This measure help quantify the contribution of predictor variables to the variance explained in the dependent variable, allowing an assessment of their significant effect. *Significance level (α)*, typically set at 0.05, defines the maximum acceptable probability of a false-positive conclusion. *Power*, which represents the probability of correctly rejecting the null hypothesis, is typically targeted at 0.80, implying an 80% chance of detecting the true effect. *Degrees of freedom*, which depends on the number of predictors, influence the sensitivity of the statistical test.

In R software, power analysis can be performed using the pwr.f2.test function, with the aforementioned parameters inputted. The output provided the power value for each sample size. If the power value is below 0.80, it indicates that the sample size may be insufficient to detect the expected effect, suggesting the need for a larger sample size. Conversely, a power value close to or exceeding 0.80 suggests that the sample size is adequate for detecting the anticipated effect.

7. TCGA lung cancer cohort transcriptome differential gene pathway analysis

The transcriptomic data of tissues was downloaded from the TCGA database that included a total of 598 cases, of which 539 were tumor samples and 59 were normal samples. The raw transcriptomic data preprocessing involved removing counts with fewer than 10 reads. Genes differential expression analysis was performed by DESeq2 software between two different groups. The genes with the parameter of false discovery rate (FDR) below 0.05 and absolute fold change (FC) > 1 were considered differentially expressed genes. Differentially expressed genes were then subjected to enrichment analysis of Kyoto Encyclopedia of Genes and Genomes (KEGG) pathways.

Supplementary Figures and Tables

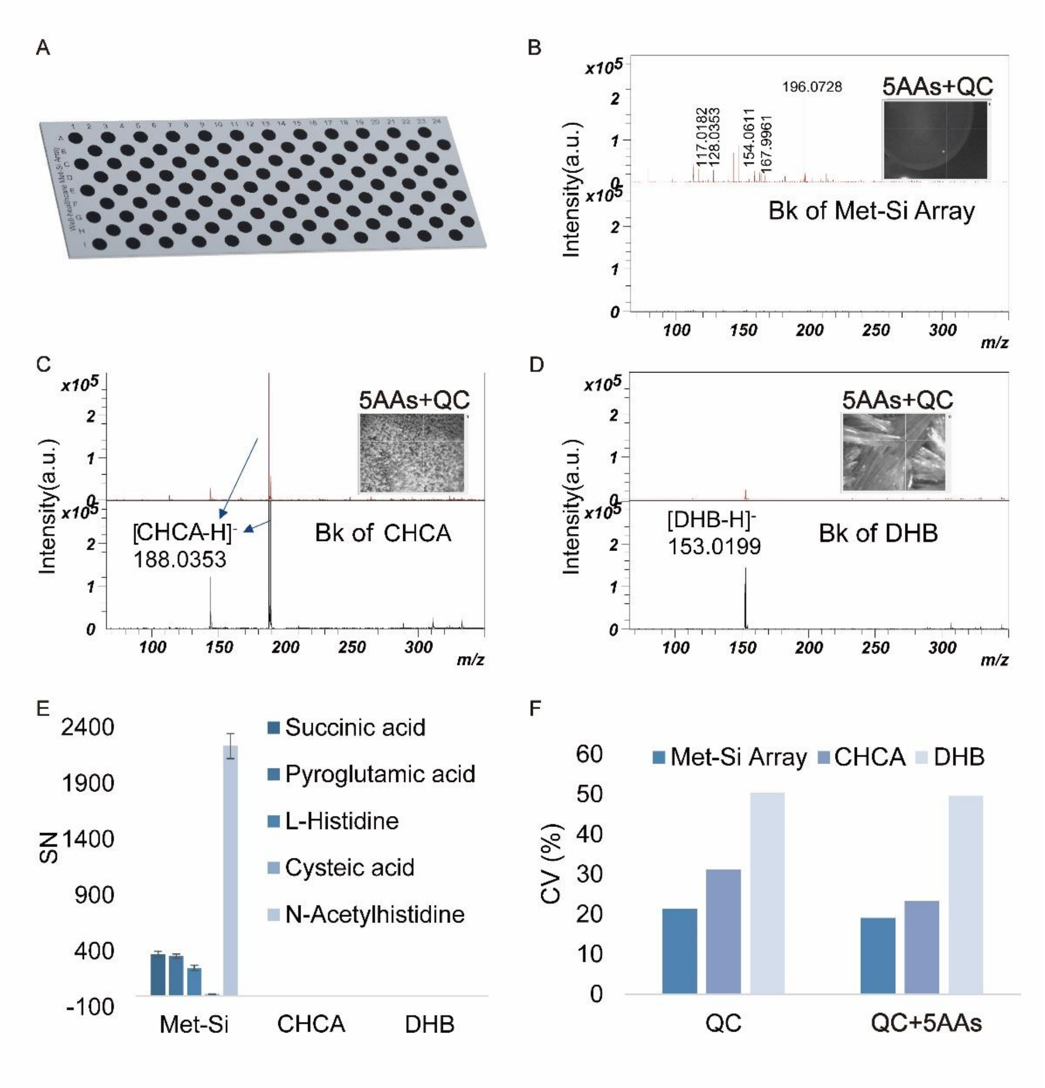

Figure S1. A schematic diagram of the Met-Si Array chip and sensitivity of MALDI-MS detection. (A) A schematic diagram of the Met-Si Array chip. (B-D) Saliva QC sample fingerprints with 5 amino acids (5AAs) mixed standards added to the internal standard and background images (below) of Met-Si Array (B), CHCA (C), and DHB (D). 5AAs included succinic acid, pyroglutamic acid, L-hisidine, cysteic acid, and N-acetylhistidine, with final concentrations of 0.033 μg/μL. The relative energy for Met-Si Array and CHCA was 71.2% while the energy of DHB was 78% caused by the high ionization threshold, which was higher compared with that of Met-Si Array and CHCA. (E) Histogram showing the comparison of peak performance of 5AAs detected on Met-Si Array, CHCA, and DHB. The fingerprints were obtained from 6 replicates. Data were presented as mean ± SD (n = 6). (F)

Histograms displaying the comparison of fingerprints consistency of QC and QC + 5AAs mixed standards detected on Met-Si Array, CHCA and DHB. The metabolic fingerprints were obtained from 6 replicates.

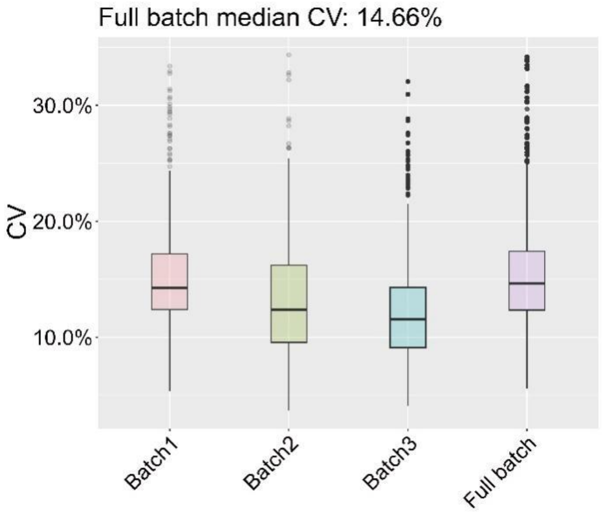

Figure S2. Stability validation of the MS LOC system. Box plots represent intra- and inter-batch CVs for QC samples across three independent batches detected on the MS LOC system (n = 96 per batch). The median CV values were 14.27% (batch 1), 11.37% (batch 2), 11.55% (batch 3), with an overall median inter-batch CV of 14.66% across all batches.

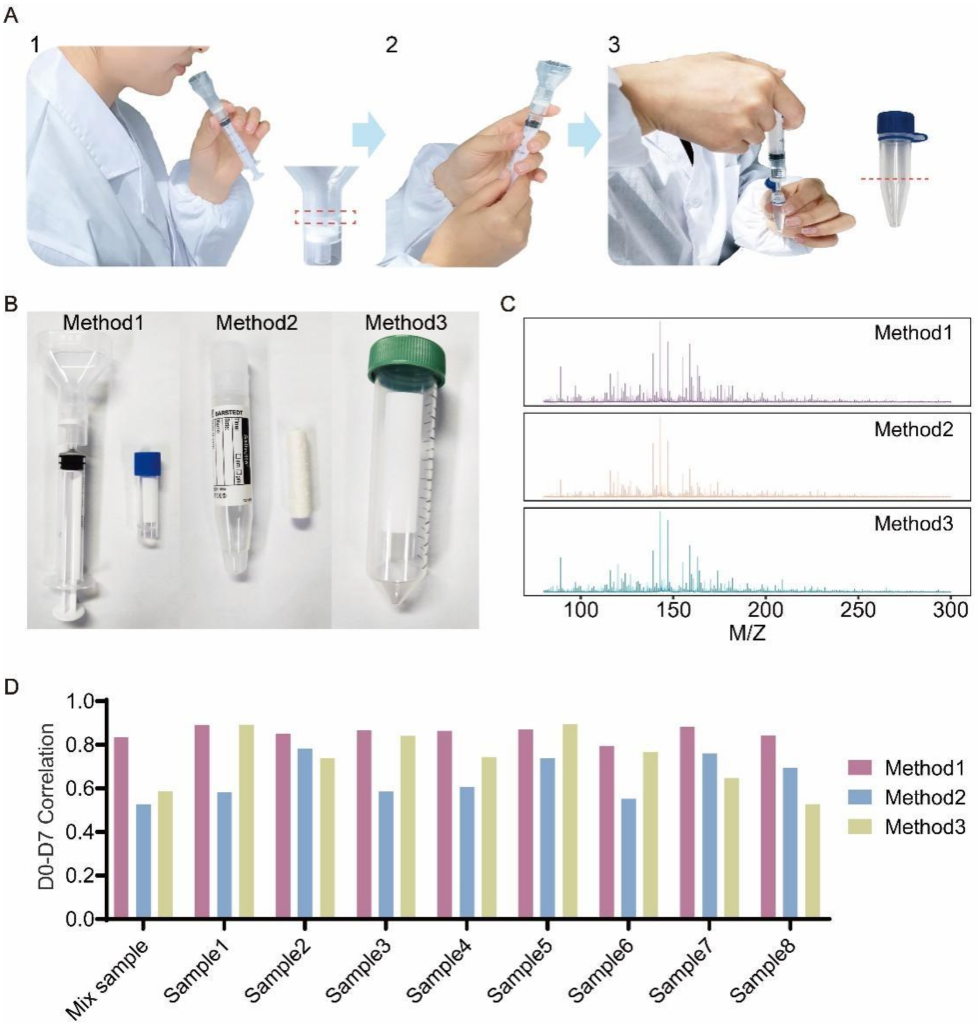

Figure S3. Three saliva collection methods and comparison of their performance. (A) Operating diagram of the Salivette device. (B) The photos of three saliva collection devices including SalivaGetin™ (Method 1, left), Salivette® (Method 2, medium) and 50 cc polypropylene tube (Method 3, right). (C) Comparison of saliva metabolomic fingerprints of the Mix sample collected using three different saliva collection devices. (D) Bar chart comparing correlation values of saliva metabolomic fingerprints on Day 0 and Day 7 at 4 °C under three different saliva collection methods. Saliva samples from eight individual volunteers (Sample 1-8) and their pooled mixture (Mix sample) were subjected to correlation analysis using R software. The correlation analysis was conducted using the Spearman method.

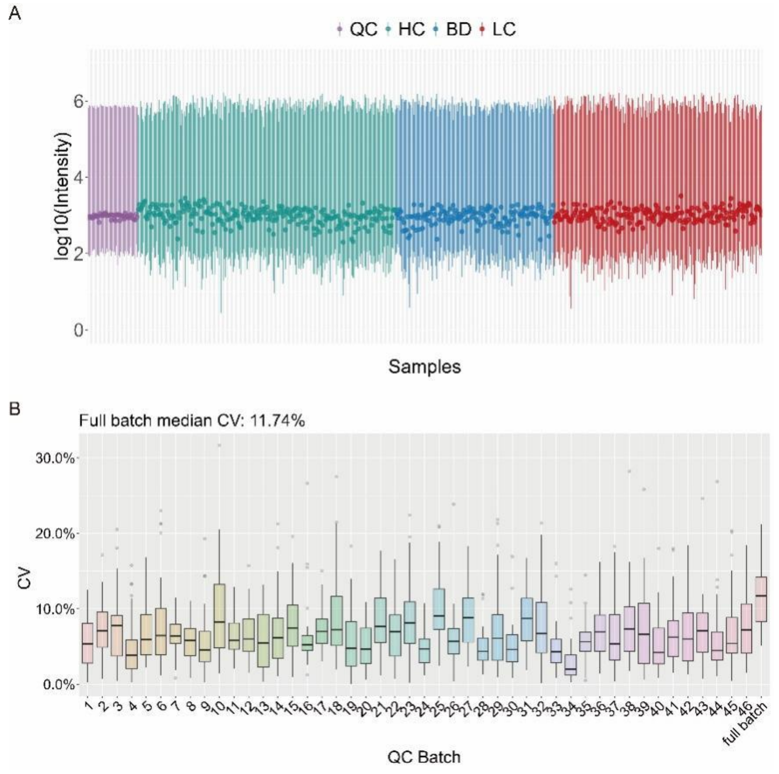

Figure S4. Evaluation of sample detection stability in the study cohort. (A) Distribution chart of MS signal intensities (log10-transformed) across study groups, including QC, HC, BD, and LC groups. (B) Box plot of CV distribution for 46 batches of QC samples, with triplicate QC samples analyzed per batch (n = 138).

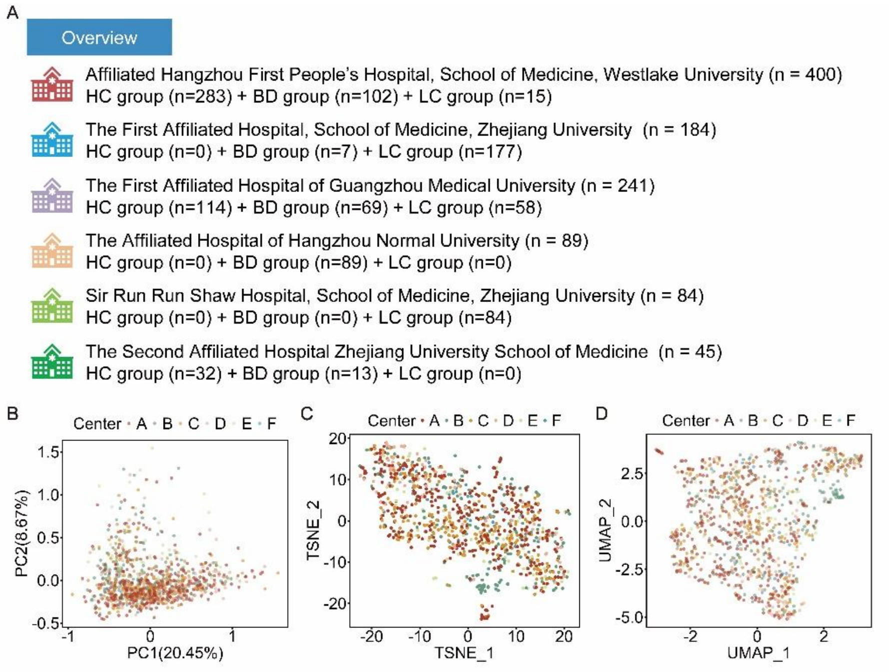

Figure S5. Sample composition and dimensionality reduction analysis across different centers. (A) Distribution of clinical samples across 6 participating centers. (B-D) Two-dimensional score plots of saliva metabolic fingerprints among samples from the six centers using PCA (B), t-SNE (C), and UMAP (D) analyses.

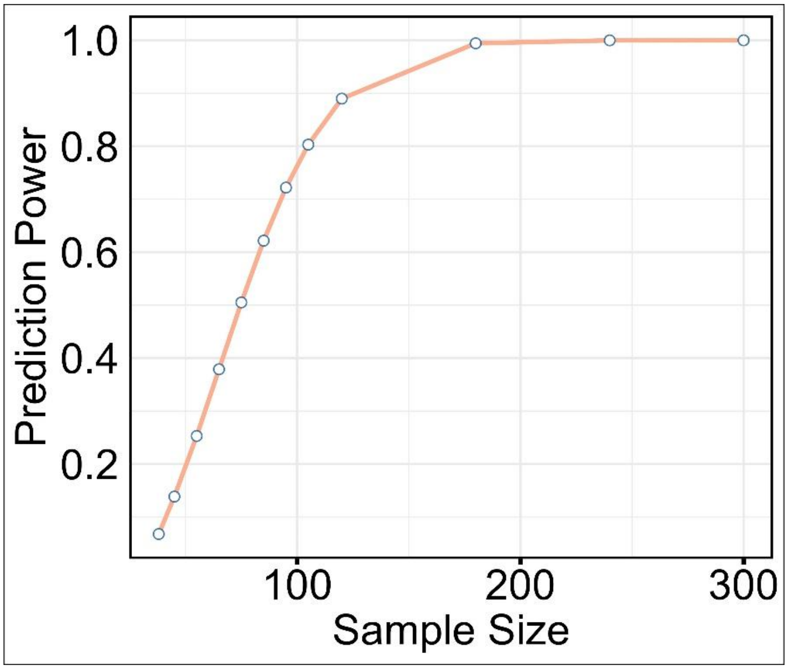

Figure S6. Power analysis of the discovery set. Power calculation using the pwr packages in R indicated that the sample size exceeding 105 could achieve a statistical power > 0.8 (α = 0.05) for predictive performance.

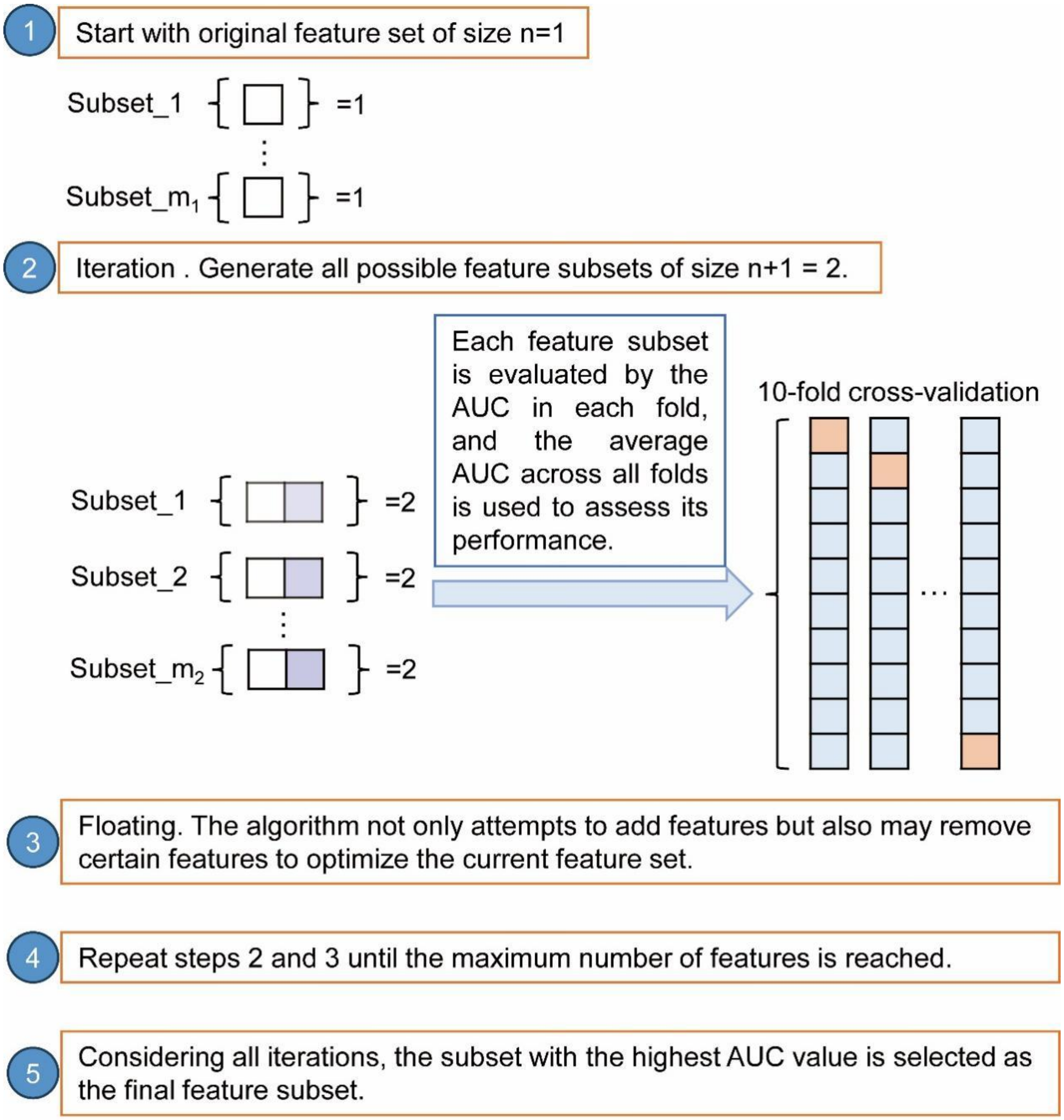

Figure S7. Illustration of SFFS for identifying feature subsets that maximize the performance of a predictive model. In this study, the candidate feature subsets were evaluated by the average AUC value from 10-fold cross-validation.

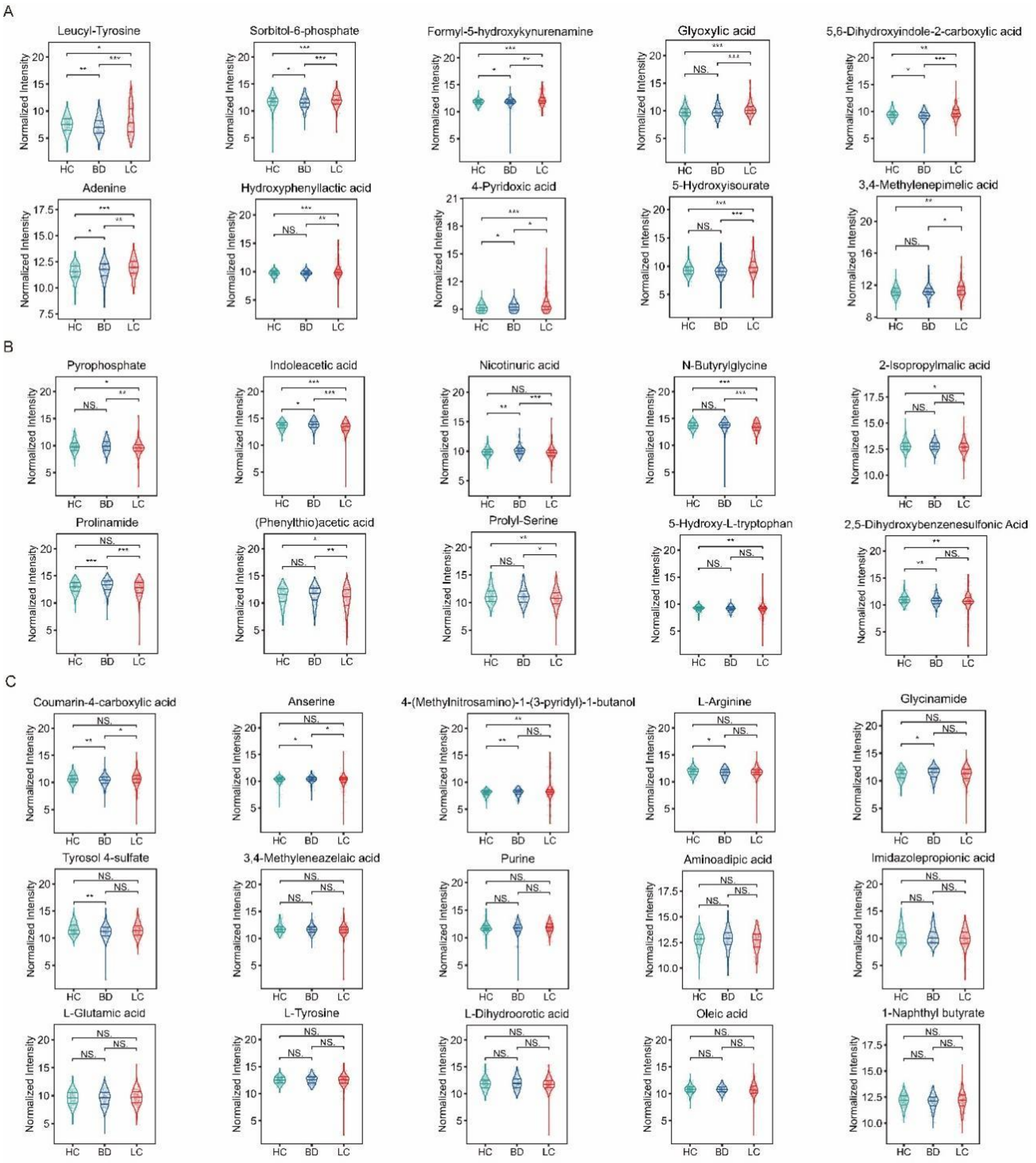

Figure S8. The distribution patterns of violin plots illustrate the relative abundance distribution of 35 model features among the HC, BD, and LC groups in the discovery set and validation set (n = 809). (A) Violin plot of metabolites significantly upregulated in the LC group compared to HC and BD groups. (B) Violin plot of metabolites downregulated in the LC group vs. HC and BD groups. (C) Violin plot illustrating abundance distribution of non-differentially expressed metabolites between LC groups and non-LC volunteers. A two-tailed Mann-Whitney U test was used for difference calculation between different groups, followed by FDR correction to obtain the p-value.adj (* < 0.05, ** p < 0.01, *** < 0.001).

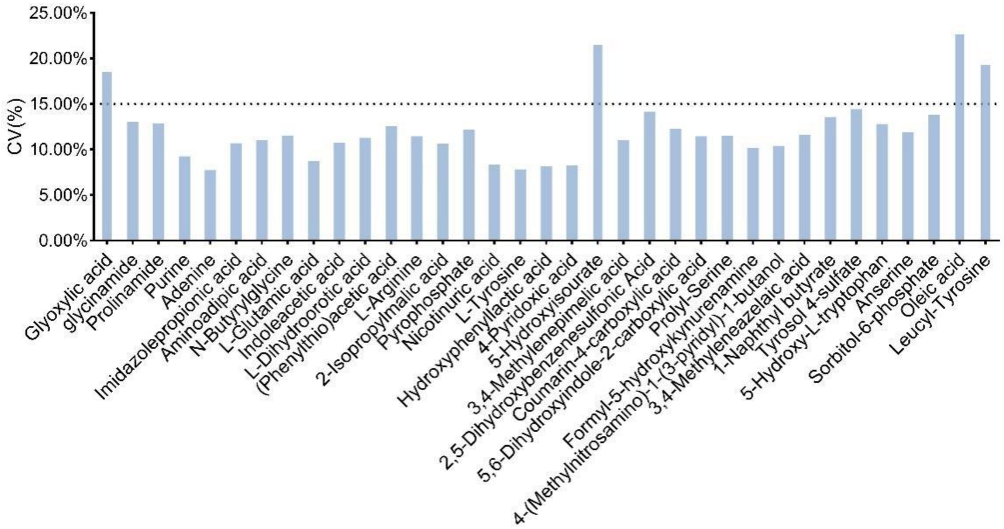

Figure S9. Analytical precision evaluation of SalivaMLD model metabolites. Bar chart displays the inter-batch CV values for 35 selected feature metabolites across 46 batches of QC samples (n = 3 per batch).

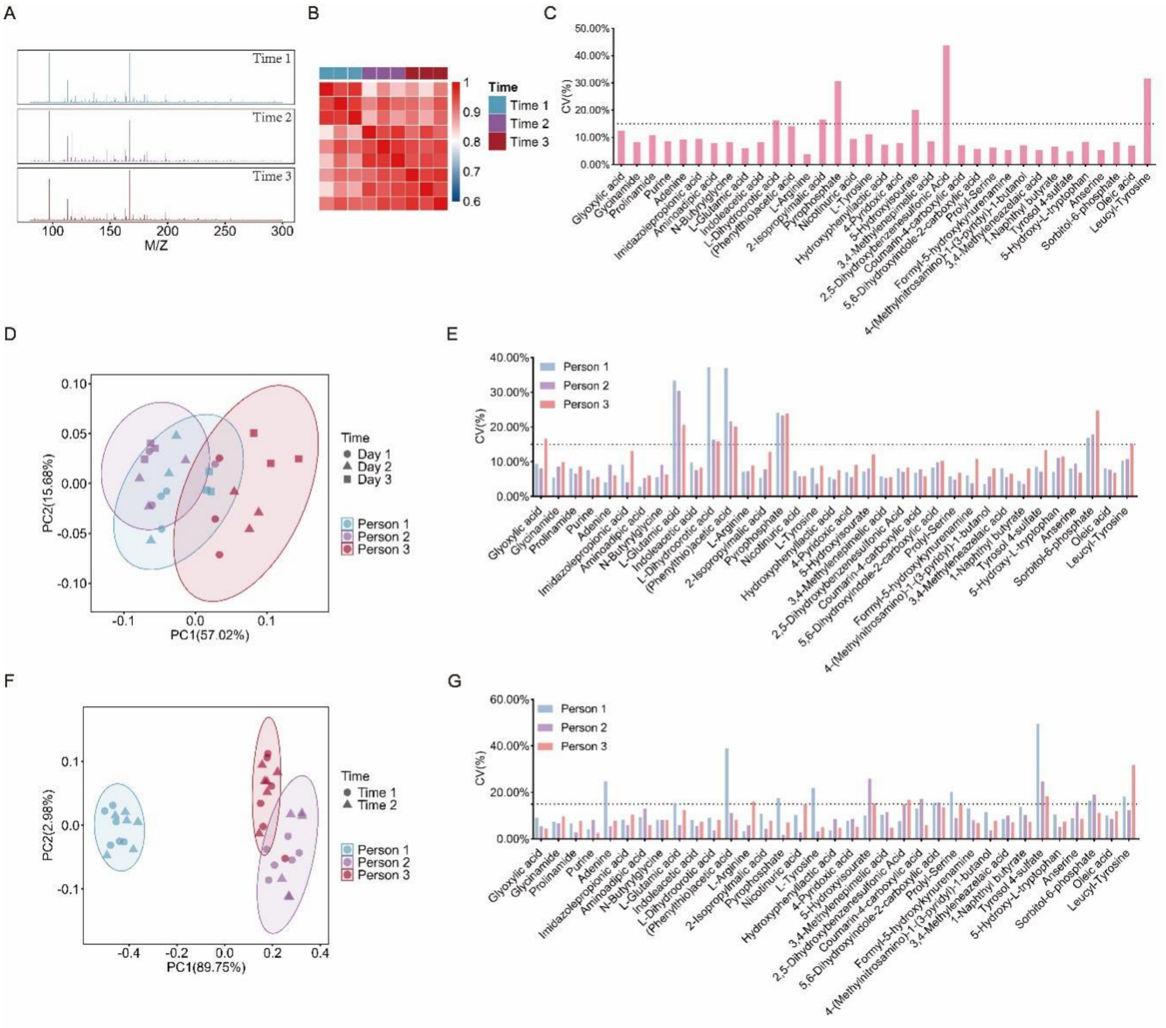

Figure S10. Influence of the temporal and dietary effect on the salivary metabolites. (A-C) Effect of different sampling time on salivary metabolic profile. (A) Averaged MS spectra of saliva samples collected from a single volunteer under three conditions on the same day. (B) Spectral correlation matrix across conditions. (C) Inter-condition CV for 35 feature metabolites. The three sampling conditions were: Time 1 (morning fasting), Time 2 (1 h post-breakfast) and Time 3 (2 h post-breakfast). For each condition, three replicate saliva samples were collected, generating a total of 9 MS spectra for CV calculation (n = 9). (D-E) Inter-day consistency of salivary metabolic profile. (D) PCA of MS spectra from three volunteers across three consecutive days (sampling at 1 h post-breakfast). (E) Inter-day CV for the 35 feature metabolites. Volunteers provided three saliva samples per day using SalivaGetin^{TM} devices. Totally, 9 MS spectra per volunteer was acquired for CV calculation (n = 9 per volunteer). (F-G) Influence of diets on salivary metabolic profile. (F) PCA of MS spectra from three volunteers under: Time 1 (fasted, day 1) and Time 2 (1 h post-breakfast, day 2). (G) Inter-day CV of 35 feature metabolites for three volunteers. These three volunteers provided two saliva samples at 9:00 am on two consecutive days using SalivaGetin^{TM} devices, with each sample

undergoing triplicate pretreatment. A total of 12 MS spectra per volunteer were used for CV calculation (n = 12 per volunteer).

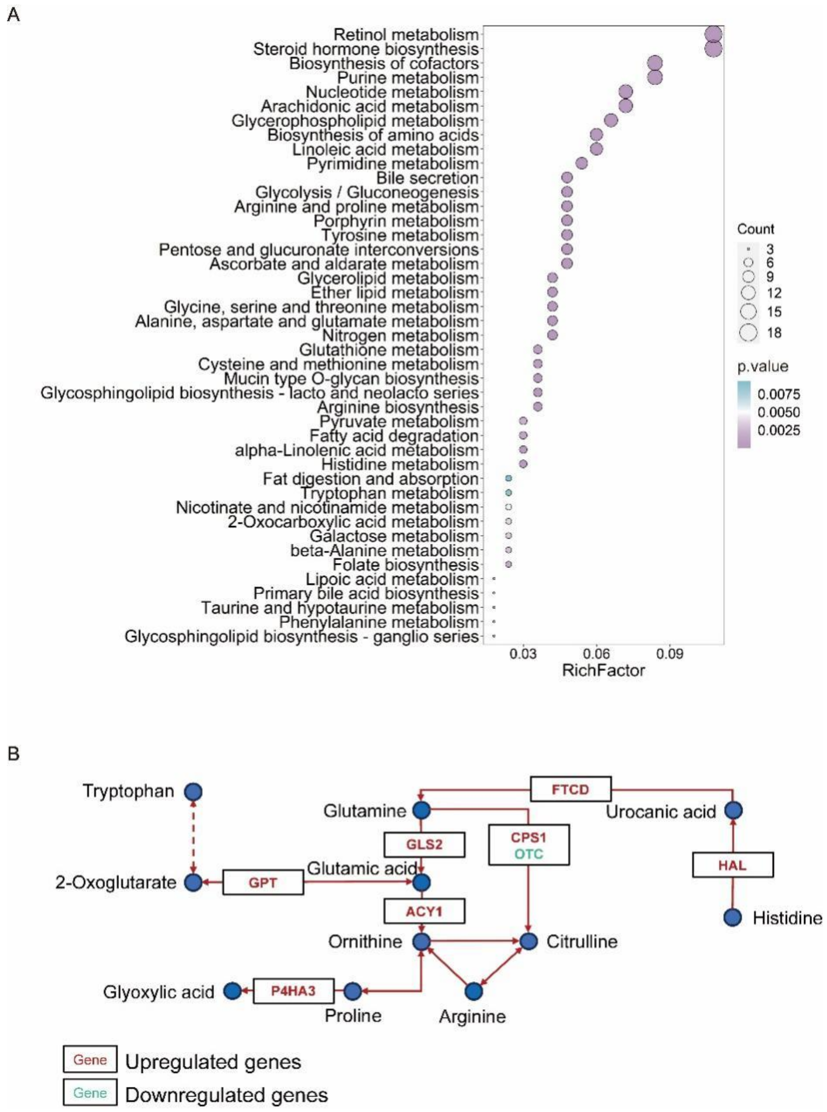

Figure S11. TCGA lung cancer cohort (n for tumor = 539, n for normal = 59) transcriptome differential gene pathway analysis. (A) Differential gene KEGG pathway enrichment analysis. The screening criteria for differential metabolic genes were $|\log_2 FC| > 2$ and p.value.adj < 0.05. (B) Metabolic pathway diagrams of differential genes in arginine and proline metabolism, tryptophan metabolism, arginine biosynthesis, and histidine metabolism.

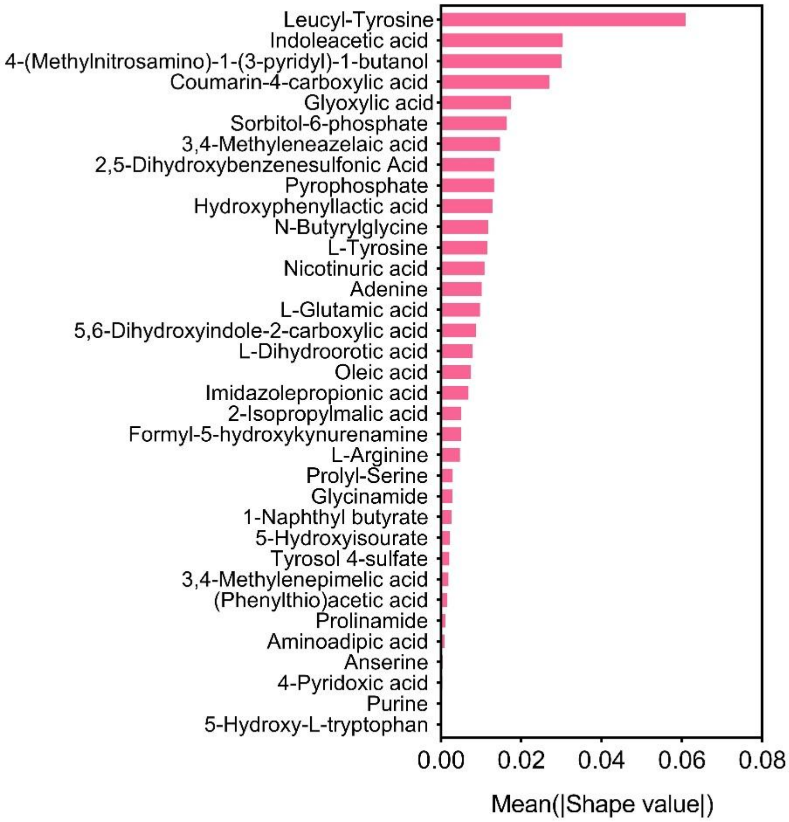

Figure S12. Bar chart of SHAP value statistics for the 35 selected features in the SalivaMLD model.

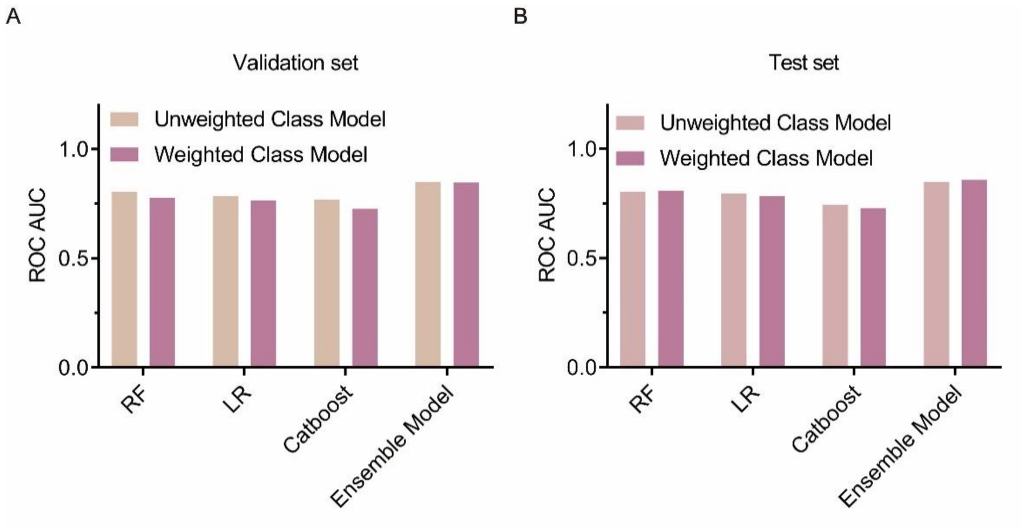

Figure S13. Comparison of model performance before and after class weighting. (A) Comparison of

ROC AUC values for the model before and after class weighting on the validation set (n = 236). (B) Comparison of ROC AUC values for the model before and after class weighting on the test set (n = 234).

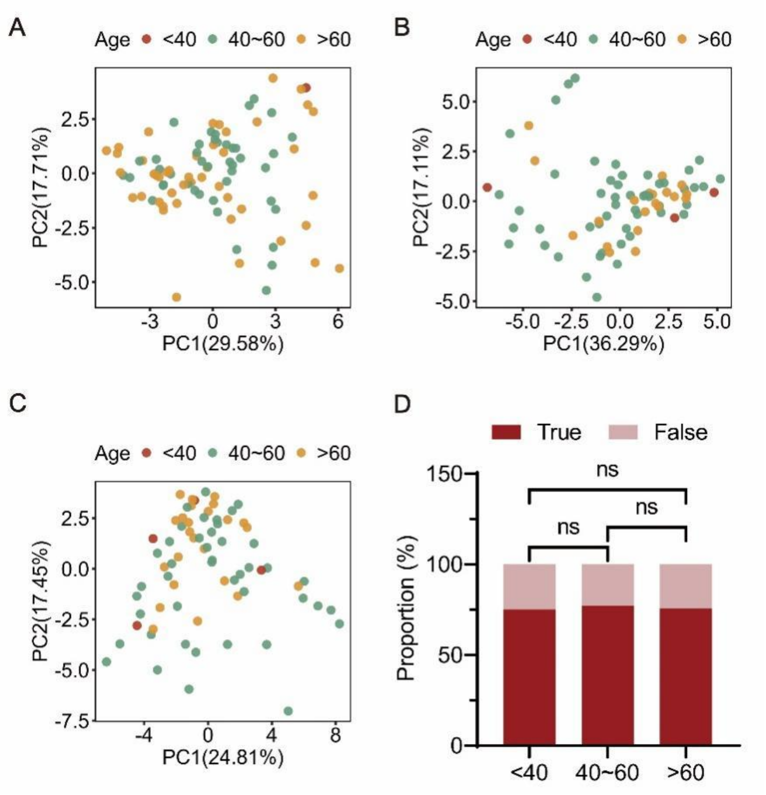

Figure S14. Age-stratified analysis of SalivaMLD performance in the test cohort. (A-C) PCA score plots of 35 model-selected metabolic features across age subgroups (< 40, 40-60, > 60 years) in the HC group (A), BD group (B), and LC group (C). (D) Stacked bar plot demonstrating age-specific prediction accuracy. Statistical significance was assessed by Chi-square test, with $p < 0.05$ considered significant, and 'ns' indicating no significance. The sample numbers for < 40, 40-60, > 60 years were 8, 136 and 90, respectively.

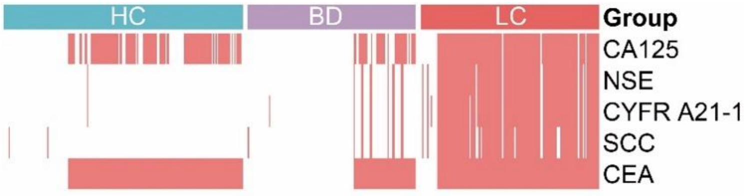

Figure S15. Data completeness of tumor biomarkers across study cohorts. Distribution profiles show

the availability of five tumor biomarkers in different sample groups, including CA125, NSE, CYFR A21-1, SCC and CEA.

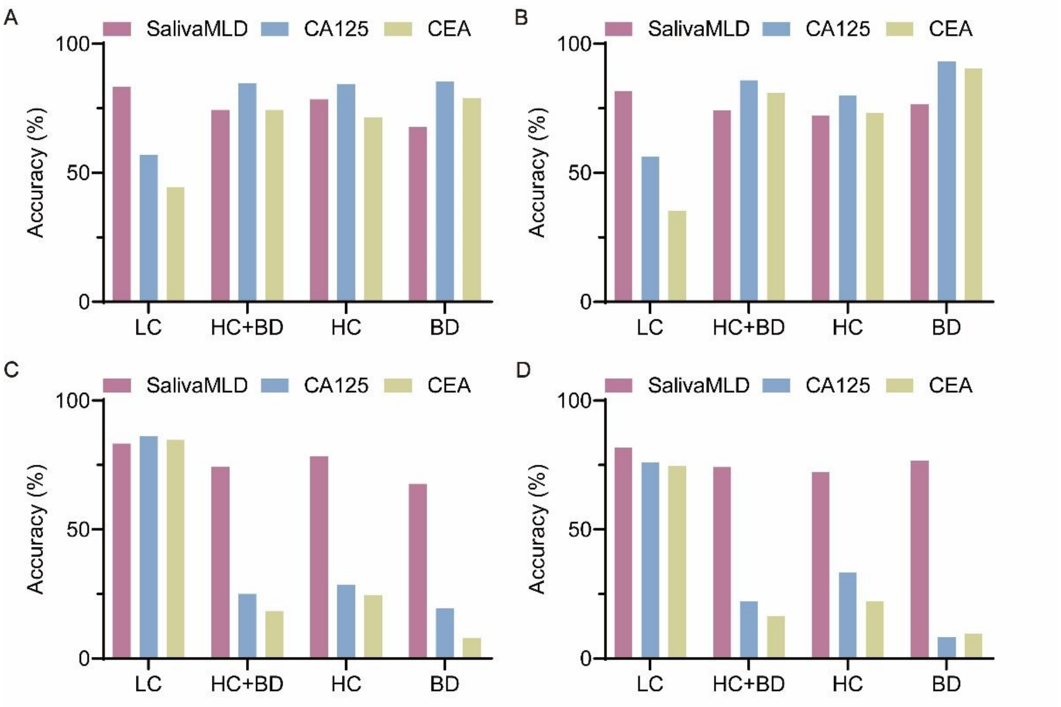

Figure S16. Comparison of the performance between SalivaMLD, CEA and CA125. (A) Under similar specificity, the accuracy of SalivaMLD, CEA, and CA125 in identifying LC, HC, and BD groups in the validation set (n = 236). (B) Under similar specificity, the accuracy of SalivaMLD, CEA, and CA125 in identifying LC, HC, and BD groups in the test set (n = 234). (C) Under similar sensitivity, the accuracy of SalivaMLD, CEA, and CA125 in identifying LC, HC, and BD groups in the validation set (n = 236). (D) Under similar sensitivity, the accuracy of SalivaMLD, CEA, and CA125 in identifying LC, HC, and BD groups in the test set (n = 234). Missing values of CEA and CA125 were filled with the median value.

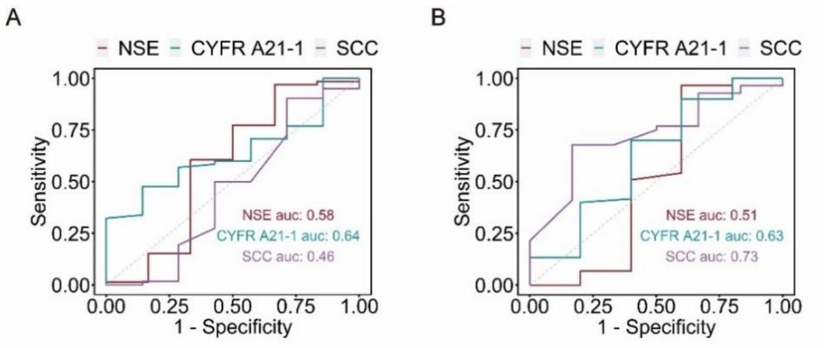

Figure S17. Diagnostic performance evaluation of tumor biomarkers. (A) ROC curves for NSE, CYFR A21-1, and SCC in the validation set (n = 236). The 95% CIs for NSE, CYFR A21-1 and SCC were [0.25-0.92], [0.46-0.82], [0.17-0.76], respectively. (B) ROC curves for NSE, CYFR A21-1, and SCC in the test set (n = 234), showing 95% CIs: NSE [0.09-0.93], CYFR A21-1 [0.31-0.95], and SCC [0.53-0.93]. Missing values were filled with the median value.

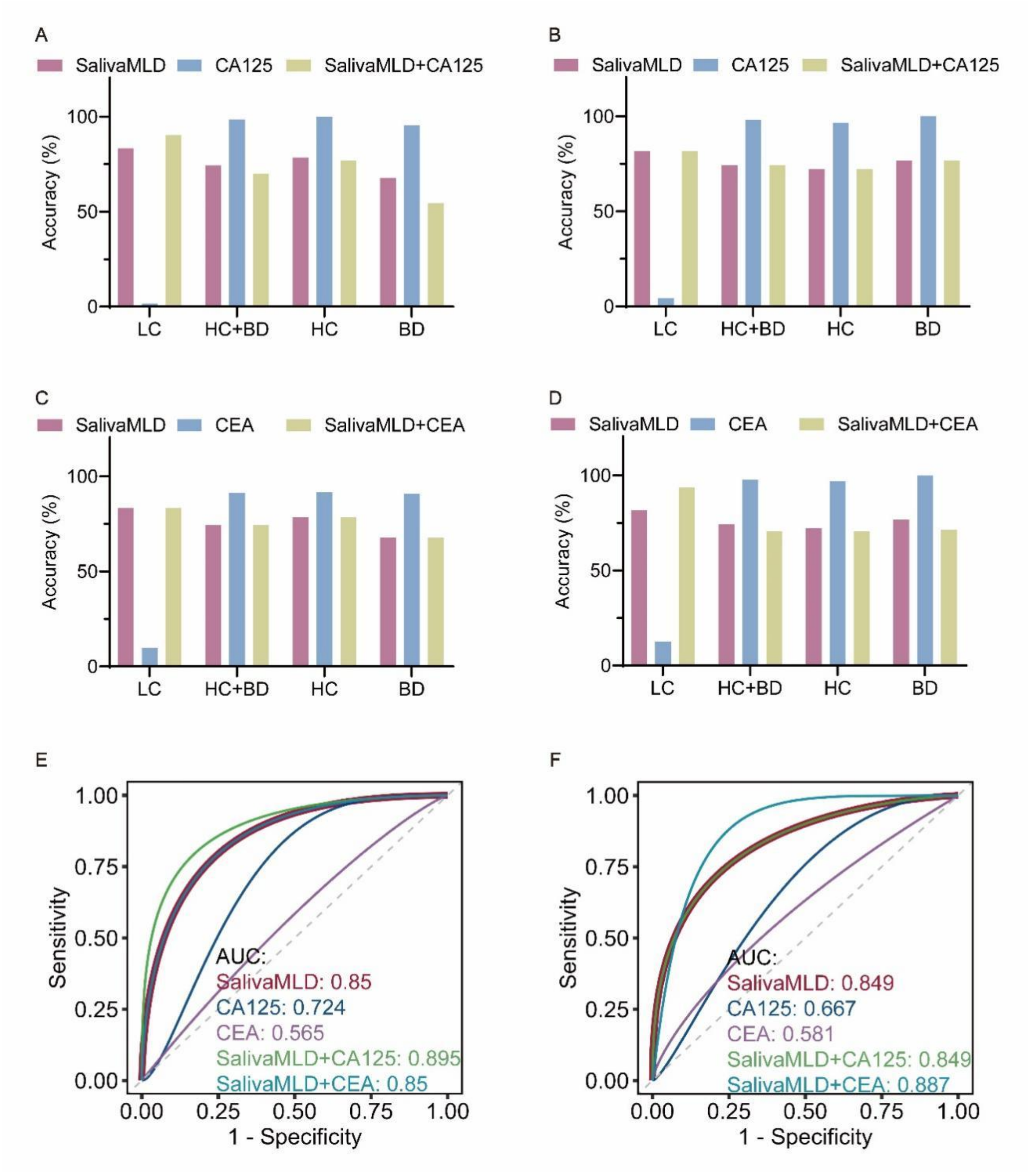

Figure S18. Comparison of the diagnostic performance between SalivaMLD, CEA, CA125,

SalivaMLD combined with CA125, and SalivaMLD combined with CEA. (A-B) The classification accuracy of SalivaMLD, CA125, and SalivaMLD combined with CA125 in identifying LC, HC and BD groups in the validation (n = 236) (A) and test (n = 234) (B) sets. (C-D) The classification accuracy of SalivaMLD, CEA, and SalivaMLD combined with CEA in identifying LC, HC and BD groups in the validation (n = 236) (C) and test (n = 234) (D) sets. (E-F) Comparison of ROC curves for SalivaMLD, CEA, CA125, SalivaMLD combined with CA125, and SalivaMLD combined with CEA in the validation (n = 236) (E) and test (n = 234) (F) sets.

Table S1. Demographic and clinical information for the study cohort stratified into discovery, validation, and test sets. Inter-group comparisons were performed between LC group and non-LC group (HC + BD) using the chi-square test, with p.values < 0.05 was considered significant (* < 0.05, ** < 0.01).

	Discovery set				Validation set				Test set			
**Total**	**HC** (n = 237)	**BD** (n = 145)	**LC** (n = 191)	**p.value**	**HC** (n = 102)	**BD** (n = 62)	**LC** (n = 72)	**p.value**	**HC** (n = 90)	**BD** (n = 73)	**LC** (n = 71)	**p.value**
**Age**				0.14				0.56				0.017*
< 40	5 (2.11%)	9 (6.21%)	11 (5.76%)		1 (0.98%)	2 (3.23%)	3 (4.17%)		1 (1.11%)	3 (4.11%)	4 (5.63%)	
40-60	121 (51.05%)	81 (55.86%)	99 (51.83%)		49 (48.04%)	34 (54.84%)	37 (51.39%)		43 (47.78%)	50 (68.49%)	43 (60.56%)	
> 60	111 (46.84%)	55 (37.93%)	81 (42.41%)		52 (50.98%)	26 (41.94%)	32 (44.44%)		46 (51.11%)	20 (27.40%)	24 (33.80%)	
**Gender**				0.0042**				0.37				0.28
Female	168 (70.89%)	80 (55.17%)	132 (69.11%)		62 (60.78%)	41 (66.13%)	39 (54.17%)		51 (56.67%)	50 (68.49%)	42 (59.15%)	
Male	69 (29.11%)	65 (44.83%)	59 (30.89%)		40 (39.22%)	21 (33.87%)	33 (45.83%)		39 (43.33%)	23 (31.51%)	29 (40.85%)	
**Smoking history**				0.049*				0.58				0.063
No	197 (83.12%)	114 (78.62%)	169 (88.48%)		79 (77.45%)	51 (82.26%)	60 (83.33%)		65 (72.22%)	61 (83.56%)	61 (85.92%)	
Yes	40 (16.88%)	31 (21.38%)	22 (11.52%)		23 (22.55%)	11 (17.74%)	12 (16.67%)		25 (27.78%)	12 (16.44%)	10 (14.08%)	
**Family history of lung cancer**				0.38				0.65				0.069
No	218 (91.98%)	128 (88.28%)	176 (92.15%)		88 (86.27%)	53 (85.48%)	65 (90.28%)		77 (85.56%)	69 (94.52%)	67 (94.37%)	
Yes	19 (8.02%)	17 (11.72%)	15 (7.85%)		14 (13.73%)	9 (14.52%)	7 (9.72%)		13 (14.44%)	4 (5.48%)	4 (5.63%)	

**Environmental exposure**				0.11				0.021*				0.38
No	200 (84.39%)	128 (88.28%)	174 (91.10%)		86 (84.31%)	56 (90.32%)	70 (97.22%)		80 (88.89%)	69 (94.52%)	63 (88.73%)	
Yes	37 (15.61%)	17 (11.72%)	17 (8.90%)		16 (15.69%)	6 (9.68%)	2 (2.78%)		10 (11.11%)	4 (5.48%)	8 (11.27%)	
**Stage of lung cancer**												
0			5 (2.62%)				4 (5.56%)				2 (2.82%)	
I			163 (85.34%)				57 (79.17%)				60 (84.51%)	
II			6 (3.14%)				2 (2.78%)				1 (1.41%)	
III			7 (3.66%)				2 (2.78%)				3 (4.23%)	
IV			2 (1.05%)				1 (1.39%)					
Unknown			8 (4.19%)				6 (8.33%)				5 (7.04%)	
**Subtype**												
LUAD			180 (94.24%)				67 (93.06%)				63 (88.73%)	
SCC			7 (3.66%)				4 (5.56%)				5 (7.04%)	
LCC			1 (0.52%)				0 (0%)				1 (1.41%)	
ASC			3 (1.57%)				0 (0%)				0 (0%)	
SCLC			0 (0%)				1 (1.39%)				0 (0%)	
Unknown			0 (0%)				0 (0%)				2 (2.82%)	

Table S2. Comparative evaluation of nine ML algorithm models after feature selection using the p-value filtering method. Univariate feature screening was performed in the validation set (n = 236) using the Mann-Whitney U test, with p.value.adj < 0.05 was considered statistically significant.

Algorithm	AUC of p-value-based models
RF	0.782
LR	0.774
Catboost	0.744
Adaboost	0.752
GBM	0.734
SVM	0.723
KNN	0.67
XGB	0.686
LightGBM	0.733

Table S3. Comparative performance evaluation of different feature across the top three models during feature selection using the SFFS method. AUC were derived from 10-fold cross-validation on the discovery set (n = 573).

	AUC value trend		
**Number of features**	**RF**	**LR**	**Catboost**
1	0.667	0.659	0.651
2	0.715	0.678	0.668
3	0.752	0.693	0.707
4	0.762	0.714	0.720
5	0.773	0.717	0.768
6	0.782	0.723	0.788
7	0.787	0.729	0.787
8	0.790	0.743	0.790
9	0.794	0.744	0.794
10	0.801	0.745	0.801
11	0.807	0.748	0.799
12	0.808	0.747	0.799
13	0.800	0.740	0.803
14	0.803	0.750	0.794
15	0.797	0.749	0.792
16	0.800	0.749	0.795
17	0.794	0.748	0.790
18	0.798	0.748	0.790
19	0.792	0.750	0.791
20	0.797	0.748	0.793
21	0.787	0.754	0.789
22	0.782	0.759	0.788
23	0.776	0.750	0.790
24	0.767	0.750	0.790
25	0.752	0.750	0.790

Table S4. Molecular information of 35 selected metabolic features for the SalivaMLD model.

Measured m/z	Model	HMDB_ID	KEGG_ID	Name	Chemical formular	Theoretical m/z	Adduction	MS2.Fragements	Delta (ppm)
72.9914	LR	HMDB0000119	C00048	Glyoxylic acid	C2H2O3	72.9931	[M-H]-	ND	-23.26
73.0408	Catboost	HMDB0062472	ND[a]	Glycinamide	C2H6N2O	73.0407	[M-H]-	ND	1.38
113.0715	LR	HMDB0253910	ND	Prolinamide	C5H10N2O	113.0720	[M-H]-	42,44,69,96,113	-4.39
119.0366	LR	HMDB0001366	C15587	Purine	C5H4N4	119.0363	[M-H]-	65,77,79,90,92,119	2.35
134.0473	LR	HMDB0000034	C00147	Adenine	C5H5N5	134.0472	[M-H]-	41,92,106,107,134	0.75
139.0518	RF	HMDB0002271	C20522	Imidazolepropionic acid	C6H8N2O2	139.0513	[M-H]-	41,95,120,121,139	3.59
142.0527	RF	HMDB0000510	C00956	Aminoadipic acid	C6H11NO4	142.0504	[M-H_2O-H]-	41,72,98,99,112,114,116,142	16.19
144.0667	RF	HMDB0000808	ND	N-Butyrylglycine	C6H11NO3	144.0666	[M-H]-	41,100,116, 126,144	0.69
146.0469	RF	HMDB0000148	C00025	L-Glutamic acid	C5H9NO4	146.0459	[M-H]-	61,78,88,104,120,130	6.83
156.0458	Catboost/LR	HMDB0000197	C00954	Indoleacetic acid	C10H9NO2	156.0449	[M-H_2O-H]-	41,96,114,138,156	5.77
157.0269	Catboost/LR	HMDB0003349	C00337	L-Dihydroorotic acid	C5H6N2O4	157.0255	[M-H]-	42,112,113,129,139	8.91
167.0191	LR	HMDB0243657	ND	(Phenylthio)acetic acid	C8H8O2S	167.0172	[M-H]-	77,81,123	11.35
173.1024	RF	HMDB0000517	C00062	L-Arginine	C6H14N4O2	173.1044	[M-H]-	114,131,156	-11.52
175.0584	RF	HMDB0000402	C02504	2-Isopropylmalic acid	C7H12O5	175.0612	[M-H]-	85.97,113,115,129,157,175	-16.00
176.9352	Catboost	HMDB0000250	C00013	Pyrophosphate	H4O7P2	176.9359	[M-H]-	78.9,158.9,176.9	-4.24
179.0434	Catboost/LR	HMDB0003269	C05380	Nicotinuric acid	C8H8N2O3	179.0462	[M-H]-	78, 108, 133,135,150,151,152,153,161	-15.67
180.0672	RF	HMDB0000158	C00082	L-Tyrosine	C9H11NO3	180.0666	[M-H]-	72,93,119,139,163	3.33
181.0531	RF/LR	HMDB0000755	C03672	Hydroxyphenyllactic acid	C9H10O4	181.0506	[M-H]-	41,107,118,135,137	13.57
182.0485	Catboost/RF/LR	HMDB0000017	C00847	4-Pyridoxic acid	C8H9NO4	182.0459	[M-H]-	108,138,152,182	14.38
183.0132	Catboost/LR	HMDB0030097	C11821	5-Hydroxyisourate	C5H4N4O4	183.0160	[M-H]-	42,112,140,166,183	-15.03
183.0621	Catboost/LR	HMDB0059730	ND	3,4-Methylenepimelic acid	C9H12O4	183.0663	[M-H]-	41,59,95,139,165	-22.68
188.9812	Catboost/LR	HMDB0245498	ND	2,5-Dihydroxybenzenesulfonic Acid	C6H6O5S	188.9863	[M-H]-	41,77,81,147,189	-27.32
189.0179	LR	HMDB0032944	ND	Coumarin-4-carboxylic acid	C10H6O4	189.0193	[M-H]-	41,145,189	-7.81

192.0268	LR	HMDB0001253	C04185	5,6-Dihydroxyindole-2-carboxylic acid	C9H7NO4	192.0302	[M-H]-	120,146,148,192	-18.06
201.0911	LR	HMDB0029026	ND	Prolyl-Serine	C8H14N2O4	201.0881	[M-H]-	41,58,113,139,160	15.01
207.0780	Catboost/RF	HMDB0012948	C05647	Formyl-5-hydroxykynurenamine	C10H12N2O3	207.0775	[M-H]-	136,162,179,207	2.41
208.1069	LR	HMDB0041809	C19574	4-(Methylnitrosamino)-1-(3-pyridyl)-1-butanol	C10H15N3O2	208.1092	[M-H]-	57,69,78,108,120,130,148,208	-10.61
211.0989	Catboost/LR	HMDB0059744	ND	3,4-Methyleneazelaic acid	C11H16O4	211.0976	[M-H]-	139,165,211	6.34
213.0918	LR	HMDB0243959	ND	1-Naphthyl butyrate	C14H14O2	213.0921	[M-H]-	127,143,185,213	-1.30
217.0117	LR	HMDB0041785	ND	Tyrosol 4-sulfate	C8H10O5S	217.0176	[M-H]-	96.9,187,217	-27.38
219.0714	LR	HMDB0000472	C00643	5-Hydroxy-L-tryptophan	C11H12N2O3	219.0775	[M-H]-	144,175,202	-27.78
239.1182	LR	HMDB0000194	C01262	Anserine	C10H16N4O3	239.1150	[M-H]-	152,166,168,193,194	13.39
261.0410	Catboost	HMDB0005831	C02810	Sorbitol-6-phosphate	C6H15O9P	261.0381	[M-H]-	78.9,96.9,139,261	10.98
281.2485	RF	HMDB0000207	C00712	Oleic acid	C18H34O2	281.2486	[M-H]-	197,221,223,225,239	-0.40
293.1597	Catboost/RF	HMDB0028941	ND	Leucyl-Tyrosine	C15H22N2O4	293.1507	[M-H]-	/	30.85

ND[a] means no data could be found.

Table S5. Comparative performance evaluation of nine ML algorithm models on the validation and test sets after feature selection using the SFFS method.

Dataset	Algorithm	ROC AUC	F1	Sensitivity	Specificity	Accuracy
Validation set	SalivaMLD	0.850	0.690	83.33%	74.39%	77.12%
	RF	0.804	0.640	77.78%	71.34%	73.31%
	LR	0.785	0.600	70.83%	71.34%	71.19%
	Catboost	0.769	0.567	68.06%	68.29%	68.22%
	GBM	0.745	0.539	66.67%	64.63%	65.25%
	AdaBoost	0.761	0.557	70.83%	63.41%	65.68%
	lightGBM	0.741	0.557	68.06%	66.46%	66.95%
	KNN	0.703	0.565	66.67%	69.51%	68.64%
	SVM	0.764	0.593	70.83%	70.12%	70.34%
	Xgboost	0.756	0.570	70.83%	65.85%	67.37%
Test set	SalivaMLD	0.849	0.678	81.69%	74.23%	76.50%
	RF	0.803	0.573	69.01%	68.71%	68.80%
	LR	0.796	0.625	77.46%	69.33%	71.79%
	Catboost	0.744	0.590	69.01%	71.78%	70.94%
	GBM	0.757	0.538	64.79%	66.87%	66.24%
	AdaBoost	0.748	0.615	73.24%	71.78%	72.22%
	lightGBM	0.784	0.611	71.83%	72.39%	72.22%
	KNN	0.648	0.503	46.07%	59.51%	58.97%
	SVM	0.798	0.594	80.28%	60.74%	66.67%
	Xgboost	0.739	0.525	67.61%	60.74%	62.82%

Table S6. Stage-specific diagnostic performance of SalivaMLD for LC patients in the validation set (n = 72) and test set (n = 71). P and N refers to the predicted positive and negative outcomes, respectively.

	Validation set				Test set			
**TNM Stages**	**P**	**N**	**Total**	**Accuracy**	**P**	**N**	**Total**	**Accuracy**
0	3	1	4	75.00%	1	1	2	50.00%
IA	42	9	51	82.35%	44	10	54	81.48%
IB	5	1	6	83.33%	4	2	6	66.67%
IIB	1	1	2	50.00%	1	0	1	100.00%
0+I+II	51	12	63	80.96%	50	13	63	79.37%
IIIA	1	0	1	100.00%	2	0	2	100.00%
IIIB	1	0	1	100.00%	1	0	1	100.00%
IV	1	0	1	100.00%	\	\	\	\
III+IV	3	0	3	100.00%	3	0	3	100.00%
Unknown	6	0	6	100.00%	5	0	5	100.00%

Table S7. Diagnostic performance of SalivaMLD in identifying LC patients with different pathological types in the validation (n = 72) and test (n = 71) sets. P and N refers to the predicted positive and negative outcomes, respectively. Lung adenocarcinoma (LUAD) included adenocarcinoma in situ (AIS), minimally invasive adenocarcinoma (MIA), and invasive adenocarcinoma (IAC).

**Dataset**		**Validation set**				**Test set**			
Pathology		P	N	Total	Accuracy	P	N	Total	Accuracy
LUAD	AIS	3	1	4	75.00%	5	1	6	83.33%
	MIA	13	4	17	76.47%	20	5	25	80.00%
	IAC	35	6	41	85.37%	20	4	24	83.33%
	Unknown	5	0	5	100.00%	7	1	8	87.50%
	Total	56	11	67	83.58%	52	11	63	82.54%
SCC		3	1	4	75.00%	4	1	5	80.00%
LCC		0	0	0		0	1	1	00.00%
ASC		0	0	0		0	0	0	
SCLC		1	0	1	100.00%	0	0	0	
Unknown		0	0	0		2	0	2	100.00%

Table S8. Comparative diagnostic performance of conventional serum biomarkers (CA125, CEA) and the SalivaMLD model in distinguishing LC, BD, and HC groups in the validation set (n = 236) and test set (n = 234) using clinical cutoff thresholds (CA125: 35 U/mL; CEA: 5.00 ng/mL).

Model	Group	Accuracy in validation set	Accuracy in test set
SalivaMLD	LC	83.33%	81.69%
	HC+BD	74.39%	74.23%
	HC	78.43%	72.22%
	BD	67.74%	76.71%
CA125	LC	1.61%	4.23%
	HC+BD	98.57%	98.16%
	HC	100.00%	96.67%
	BD	95.45%	100.00%
CEA	LC	9.68%	12.50%
	HC+BD	91.43%	97.75%
	HC	91.67%	97.06%
	BD	90.91%	100.00%
SalivaMLD + CA125	LC	90.32%	81.69%
	HC+BD	70.00%	74.23%
	HC	77.08%	72.22%
	BD	54.55%	76.71%
SalivaMLD + CEA	LC	83.33%	93.75%
	HC+BD	74.39%	70.79%
	HC	78.43%	70.59%
	BD	67.74%	71.43%

Table S9. Comparative diagnostic performance of conventional serum biomarkers (CA125, CEA) and the SalivaMLD model in distinguishing LC, BD, and HC groups in the validation (n = 236) and test (n = 234) sets when the thresholds of CA125 and CEA were set at similar specificity or similar sensitivity compared to the SalivaMLD model.

		Similar specificity		Similar sensitivity	
**Model or index**	**Group**	**Accuracy in validation set**	**Accuracy in test set**	**Accuracy in validation set**	**Accuracy in test set**
SalivaMLD	LC	83.33%	81.69%	83.33%	81.69%
	HC+BD	74.39%	74.23%	74.39%	74.23%
	HC	78.43%	72.22%	78.43%	72.22%
	BD	67.74%	76.71%	67.74%	76.71%
CA125	LC	56.94%	56.34%	86.11%	76.06%
	HC+BD	84.76%	85.89%	25.00%	22.09%
	HC	84.31%	80.00%	28.43%	33.33%
	BD	85.48%	93.15%	19.35%	8.22%
CEA	LC	44.44%	35.21%	84.72%	74.65%
	HC+BD	74.39%	80.98%	18.29%	16.56%
	HC	71.57%	73.33%	24.51%	22.22%
	BD	79.03%	90.41%	8.06%	9.59%
